# Simulating the Interacting Effects of Intraspecific Variation, Disturbance, and Competition on Climate-Driven Range Shifts in Trees

**DOI:** 10.1371/journal.pone.0142369

**Published:** 2015-11-11

**Authors:** Emily V. Moran, Rhys A. Ormond

**Affiliations:** School of Natural Sciences, University of California Merced, Merced, California, United States of America; Technical University in Zvolen, SLOVAKIA

## Abstract

Climate change is expected to favor shifts in plant distributions; some such shifts are already being observed along elevation gradients. However, the rate of such shifts may be limited by their ability to reach newly suitable areas and by competition from resident species. The degree of local adaptation and genetic variation may also play a role in the interaction between migrants and residents by affecting relative fitness. We used a simulation model to explore the interacting effects of dispersal, fecundity, disturbance, and genetic variation on range-edge dynamics between a pair of demographically similar tree species. Ideal climate for an individual is determined by genotype. The simulated landscape undergoes an 80-year period of climate change in which climate bands shift upslope; subsequently, climate is held constant for 300 years. The presence of a high-elevation competitor caused a significant lag in the range shift of the low-elevation species relative to competition-free scenarios. Increases in fecundity and dispersal distance both helped to speed up the replacement of the high-elevation species by the low-elevation species at their range boundary. While some disturbance scenarios facilitated this transition, frequent canopy disturbance inhibited colonization by removing reproductive adults and led to range contractions in both species. Differences between dispersal scenarios were more pronounced when disturbance was frequent (15 vs. 25 year return interval) and dispersal was limited. When the high-elevation species lacked genetic variation, its range was more-easily invaded by the low-elevation species, while a similar lack of variation in the low-elevation species inhibited colonization—but only when this lack of variation decreased the fitness of the affected species near the range boundary. Our model results support the importance of measuring and including dispersal/fecundity, disturbance type and frequency, and genetic variation when assessing the potential for range shifts and species vulnerability to climate change.

## Introduction

Climate change is driving range shifts along both latitudinal and elevation gradients as species disperse into newly suitable areas and “trailing edge” populations go extinct [1–4]. In plants, migration rate depends on both the ability of seeds to reach newly suitable areas and the establishment of migrants given factors such as competition [3,5,6]. While forest trees have decadal generation times, they generally exhibit high genetic diversity and produce large numbers of offspring, which could enable strong responses to selection [7,8]. Range shifts and local adaptation occurred simultaneously in forest trees following the end of the last glacial period [9]. However, given rapid predicted rates of contemporary climate change, lags in both migration and adaptation are expected [3,10–12]. Loarie et al. [13] estimated that species in montane areas would have to shift their ranges upward at a rate of 10–700 m/year in order to match temperature change under the A1B emission scenario, while in flatter areas shifts of 0.1–10 km/year may be required. Recent studies have demonstrated that shifts in the mean elevation of plant species along elevation gradients (usually in the range of 2.5–3.5 m/year) are already occurring due either to low-elevation mortality increases, recruitment increases in the upper part of the range, or both [14–17]. In some cases these shifts have been linked directly to temperature change [18]. However, evidence for shifts in the upper range limit, suggesting colonization of newly suitable territory beyond the original range, is still often lacking.

Classic correlative species distribution models do not take into account dispersal limitations, competitive interactions, or the potential for evolution [3,19]. Recognizing this, ecological modelers have begun to explore the impacts of these factors on climate-driven distribution changes [20]. Dispersal limitation has been widely recognized as an important factor in range shift speed, and is incorporated in most demographically-based models [6,21–27]. Because of the competitive effect of adult trees on seedlings, disturbances also play an important role in forest dynamics [28–30]. Adults trees tend to tolerate unfavorable climate better than seedlings, and may not exhibit significantly elevated mortality until a more substantial climate shift has occurred [31,32], so disturbance or increased mortality of resident adults may facilitate faster range changes in response to climate change [33,34]. For instance, a 2.07–2.7 m/year shift of the boreal-hardwood forest ecotone in Vermont’s Green Mountains has been attributed to an increase in low-elevation species recruitment that was made possible by an increase in mortality in two dominant high-elevation species [34]. A simulation study [10] found that evolution of bud phenology traits in trees lagged behind their optimum values as climate shifted, and that this lag was reduced by higher adult mortality rates. However, this model implicitly assumed the formation of single-tree gaps, which are often too small to promote much recruitment in shade-intolerant species [35]. Larger-scale disturbances, such as those caused by fire, might provide more opportunities for recruitment of better-adapted individuals.

Previous theoretical models have shown that competition can slow range shifts under climate change [5,21,36–38], that the persistence of cold-adapted adults can block the spread of warm-adapted individuals [39], and that disturbance can interact with competition to affect range dynamics [21]. Several models have addressed the landscape-scale effects of some of these factors on climate responses in trees. A model for European beech found that multilocus genetic variation in budburst phenology and stomatal conductance affected range dynamics via the potential for local adaptation, but did not examine interspecific competition or disturbance [40]. The Phenofit model has shown that variation in dispersal ability and in growth and fecundity responses between species and populations can have important effects on the potential for range shifts, but did not include competition or the effects of gene flow between currently locally adapted populations [26]. The TreeMig model has incorporated dispersal ability and competition, but not genetic variation. While disturbance, interspecific competition, and intraspecific genetic variation are all recognized as important for climate change responses [3,8,41–43], no model to date has examined how all three factors interact at range boundaries.

We developed a two-species simulation model incorporating genetic variation at a single locus to investigate the potential for interactions between interspecific competition, dispersal ability, disturbance frequency, and intraspecific variation in climate responses to affect range-shift responses to climate change. The two species represent a high-elevation species and a low-elevation species that are otherwise similar in their life history and demography. It is not uncommon in montane areas to encounter two closely related species that are similar in many life-history traits, but have distinct elevation distributions. For instance, in the Sierra Nevada of California, such low-elevation/high-elevation species pairs include white fir (*Abies concolor*) and red fir (*A*. *magnifica*); sugar pine (*Pinus lambertiana*) and western white pine (*P*. *monticola*); and ponderosa pine (*P*. *ponderosa*) and Jeffrey pine (*P*. *jeffreyi*) [44,45]. The same can also be true along latitudinal gradients, though the distances involved are greater. Model variants differ in seed and pollen dispersal distance, fecundity, disturbance, and/or intraspecific variation. We show that all of these factors interact to affect range shift speed.

## Methods

This model description follows the ODD (Overview, Design concepts, Details) protocol [46,47]. The model was written in R [48]. Code for the baseline model is given in S1.

### Purpose

This model is intended to simulate demographic responses to climate and competition for a system in which there are both between-species and within-species differences in climate responses, so that interactions between dispersal, competition, genetic variation, and disturbance during range shifts can be investigated.

### Entities, State Variables, and Scales

**Fig 1:**
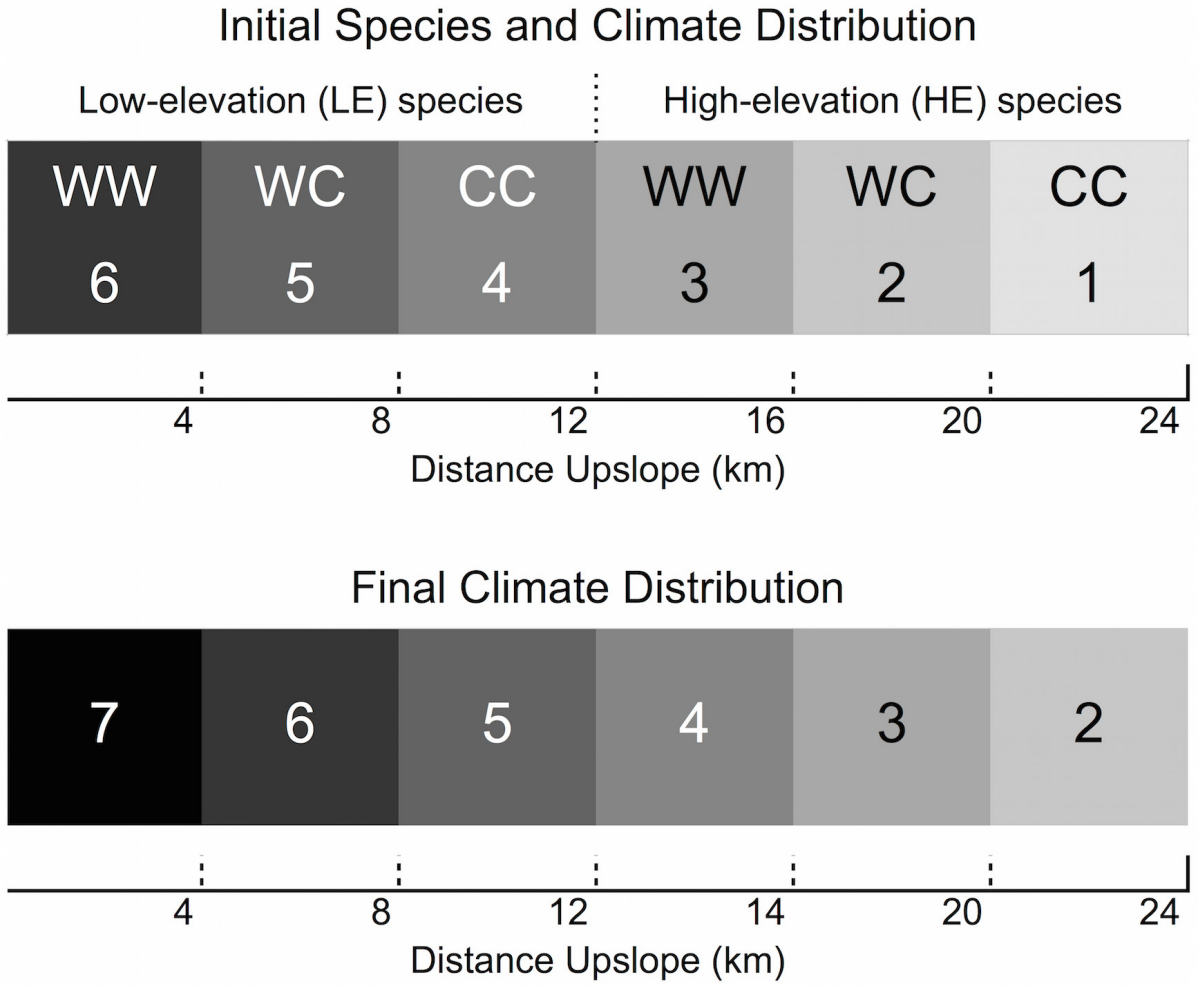
Initial distribution of species, genotypes, and climate across the model landscape (top). W indicates “warm climate allele” and C “cold climate allele” shading represents the ideal climate for each genotype. Initial climates range from 6 (hottest) to 1 (coldest). Final climate distribution after the 80-year period of climate change (bottom) ranges from 7 to 2.

The simulation landscape consists of 50 x 50m cells arranged into a 20 x 480 cell rectangular grid (1 km high x 24 km long). The climate gradient is initially represented by 6 vertical bands, 80 cells (4km) wide, representing climates 6 (the warmest) on the left to 1 (the coldest) on the right (Fig 1). Moving from left to right on the grid is equivalent to moving up a mountain from warmer to colder climates. Moving up and down the grid within the same climate band is equivalent to moving across-slope.

Trees may belong to one of two species—low elevation (LE) and high elevation (HE). Within each species, each individual is either genotype WW, WC, or CC, which have maximal performance in the warmest (climate 6 for LE, climate 3 for HE), middle (climates 5 and 2), or coldest (climates 4 and 1) part of the species range, respectively. Trees are categorized according to their size stage: seedlings (<0.5 cm tall), saplings (>0.5 m tall, <5 cm diameter-at-breast-height (DBH)), small (5–29 cm) trees, medium (30–69 cm) trees, and large (70–100 cm DBH) trees. The distribution of trees with a given species-genotype combination (e.g. LE-CW) is represented by a matrix. The number of rows is equal to the number of cells in the grid, and the number of columns is equal to the number of size classes. A similar set of 6 matrices represents the number of seeds present in each cell belonging to each species-genotype combination.

The basal area of trees of each size class present in each cell is calculated based on average basal areas for each class of 0.0001, 0.003, 0.07, 0.14, and 0.5 m^2^. The carrying capacity of each cell is 18 m^2^ of basal area. Both species have the same maximum probabilities of germination, survival in a given size class, and transition between size classes. They also have the same maximum fecundity (seed production) and pollen production. These probabilities and reproductive parameters are modified according to the basal area of larger individuals within the cell and/or the climate of the cell. Both species are assumed to suffer the same effect of competitor basal area. The species have the same probability distributions for seed and pollen dispersal, which are not altered by any other variables.

We created model variants to investigate the effect of modified parameters on range shifts (Table 1). The baseline model (code given in S1 File and parameter values in S2 File) and each model variant were run ten times each through the climate sequence to estimate variance in outcomes. These variants were also combined to explore the interaction between these different modified parameters: No high elevation species plus long dispersal for LE (NH_L); low genetic diversity and wide tolerance in HE plus simple disturbance (HNW_SD); low genetic diversity and wide tolerance in HE plus long dispersal (HNW_L); simple, canopy, or understory disturbance with a 25 rather than 15 year return interval (SD_25, CD_25, UD_25); simple, canopy or understory disturbance with short dispersal (SD_S, CD_S, UD_S); simple, canopy, or understory disturbance with long dispersal (SD_L, CD_L, UD_L); simple disturbance with high fecundity (SD_HF); simple disturbance with high fecundity and short dispersal (SD_HF_S);

**Table 1:** Model variants.

Model Variant	Abbr.	Description
Base	Base	Baseline model with no modifications.
Simple disturbance	SD	Cell-sized disturbance with an average return time of 15 years. Individuals in disturbed cells have a 30% chance of survival.
Canopy disturbance	CD	Similar to SD but survival probability differs among age classes as follows: seedlings 30%, saplings 15%, small trees 5%, medium trees 10%, large trees 20%.
Understory disturbance	UD	Similar to SD but survival probability differs among age classes as follows: seedlings 10%, sapling 15%, small trees 35%, medium trees 70%, large trees 95%.
25yr disturbance return	25	Disturbance with an average return time of 25 years.
Longer dispersal	L	Dispersal distances increased by 50%, to an average of 75 m for seed and 360 m for pollen.
Shorter dispersal	S	Dispersal distances reduced by 50%, to an average of 25 m for seeds and 120 m for pollen.
High fecundity	HF	Trees produce 4x as much viable seed.
No HE species	NH	LE species only, no HE species modeled.
No LE genetic variation, broad tolerance	LNW	LE species has a single genotype that performs best in climate 5 but has broader tolerances (Fig 2 top left).
No HE genetic variation, broad tolerance	HNW	Similar to LNW but HE rather than LE has a single genotype with broader tolerances with an optimum in climate 2 (Fig 2 top right).
No HE genetic variation, narrow tolerance	HNN2 HNN3	HE has a single genotype that performs best in either climate 2 or 3, with performance declining away from this optimum identically to individual genotypes in the baseline model (Fig 2 bottom left and right).
Increased large tree survival	LTS	Maximum large tree survival probability increased from 0.98 to 0.992.

### Process overview and scheduling

The model uses annual time-steps. In each simulation, the model is first run for 150 years under the initial climate conditions in order to allow the tree populations to reach a stable size distribution (S3 File), starting from the initial tree numbers described below. Subsequently, the climate gradient on the simulated landscape shifts by one cell (50 m) per year for 80 years. At the end of this period of change there are 6 vertical bands, each 80 cells wide, representing climates 7 to 2, with the coldest climate (1) having disappeared from the landscape. This eliminates 1/3 of the optimum habitat for the HE species, and introduces a new sub-optimal climate (7) into the LE species’ original range. The model continues to run for a further 300 years under the new stable conditions.

In each time-step, the model goes through the following processes: 1) Shifting of the climate gradient (if in the climate shift time period), 2) Calculation of the basal area of each size-class within each cell, 3) Germination of seeds, in which the resulting seedlings are added to the number of seedlings (of the corresponding genotype) in each cell, 4) Mortality, 5) Reproduction: first pollen production, then pollen dispersal, then seed genotypes production, then seed dispersal disperse, 6) Transitions to the next stage class.

### Design concepts

#### Basic Principles

The species in this model are roughly based on ponderosa and Jeffrey pine, which in the central Sierra Nevada occupy elevations from 914–1,981 m and 1,676–2,591 m, respectively [49]. These species are morphologically similar with similar life histories [44] and both exhibit local adaptation along elevation gradients [50]. Some recent data suggest that Jeffrey pine is experiencing increases in mortality at low elevations and in recruitment at higher elevations [2,18]. Justifications for parameter values and process order are given in S2 File.

**Fig 2:**
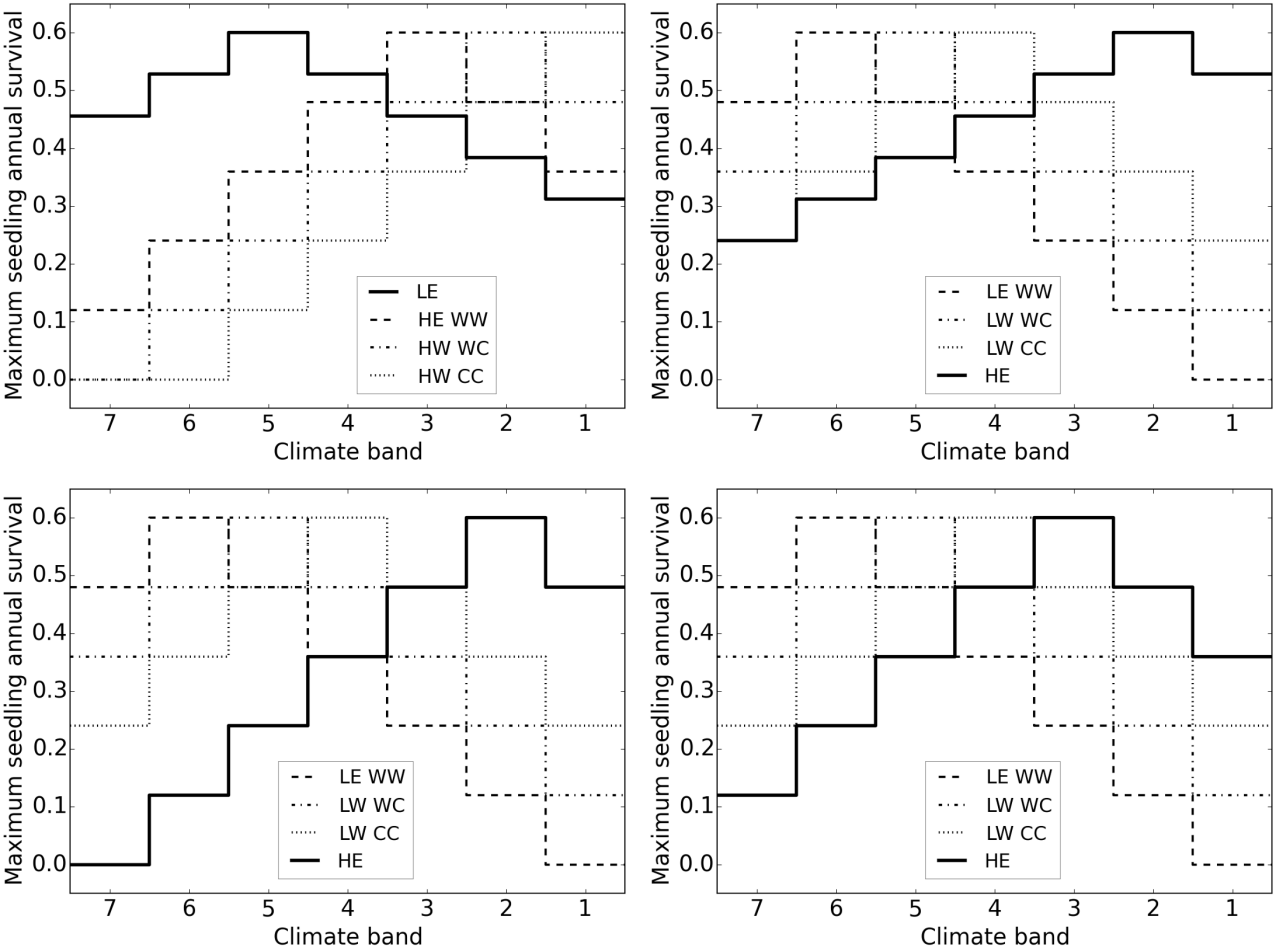
Model variants with no genetic diversity in one species (dark line) showing seedling survival as an example of patterns in demographic rates across climate bands. Top left: LE—broad tolerance, optimum climate 5. Top right: HE—broad tolerance, optimum 2. Bottom left: HE—narrow tolerance, optimum 2. Bottom right: HE—narrow tolerance, optimum 3.

Both HE and LE in this model have up to 2 alleles at a single locus that determine their response to the environment (Fig 2). While in most species many loci are involved in trait variation along ecological gradients [3,51], this simplified model of local adaptation is sufficient for the initial exploration of how local adaptation, dispersal, and disturbance interact to affect range shifts.

#### Emergence

The range dynamics emerge from the various deterministic and stochastic population processes included in the model.

#### Interaction

Interactions are not directly modeled. Rather, the survival and reproduction of a particular species or genotype depends on its demographic parameters, as shaped by climate and/or basal area of neighbors, relative to the other individuals in the cell.

#### Stochasticity

Survival and transition to the next size class are stochastic, as is disturbance in the models that include it. Germination and dispersal are deterministic, in the sense that the probability of an event (germinating or dispersing x distance) is simply multiplied by the number of seeds or pollen grains involved to calculate the number of germinants or dispersers rather than by drawing this number from a distribution.

#### Observation

Cell occupancy was calculated at the end of the initial stable period (year 0), after 80 years of climate change (year 80), and after 300 years in the new stable climate (year 380) based on adult individuals (small, medium, and large trees). Given the high juvenile mortality rate, seedling or sapling presence is no guarantee of successful population establishment. For analysis, we calculated the number of cells in which the W allele’s frequency in adults is >65%, 25–65%, and <25%, which corresponds well to areas dominated by WW, WC, or CC individuals. These values in turn were used to compare the existing distribution of species and genotypes to the expected equilibrium distribution after climate change (shown as bar “EQ” in the figures). We primarily used the range occupied by different species and genotypes at 300 years after climate change to evaluate differences between model variants. For select model variants, we also let the simulation run for 5000 years after climate change to estimate the time until the new equilibrium was reached.

Due to competition from LE, HE is eventually excluded from the area of its range that shifts to climate 4. LE can persist in climate 7 areas because there is no lower-elevation species with higher fitness in this simulation (Figure A in S5 File). However, we refer in the figures below to both climates >3 occupied by HE and climate 7 areas occupied by LE as “extinction lag” because in nature LE would likely be displaced from this suboptimal area by a third species. Climate 4–6 areas not occupied by LE and climate 2–3 areas not occupied by HE were considered to represent a colonization lag, as they are within the preferred climate range of the species and thus should be occupied if there is sufficient colonization of suitable open cells.

### Initialization

Initially, there are 5 seedlings, 20 saplings, 18 small trees, 6 medium trees, and 2 large trees of the optimum genotype for the climate established in each cell. After the first 150 years in the initial stable climate, the size distribution stabilizes at an average of 276.7 seedlings, 49.9 saplings, 20.3 small trees, 10.8 medium trees, and 10.1 large trees per cell in the baseline model.

### Submodels

#### Calculation of seed and pollen dispersal probabilities to each cell

Both seed and pollen were assumed in the baseline model to have fat-tailed 2-Dt dispersal kernels, a probability distribution that describes many tree species well [52]. In the baseline model, dispersal parameters were chosen to yield an average dispersal distance of 50 m for seed and 240 m for pollen. Using a grid centered on a source tree, we simulated 200,000 dispersal events. This procedure is used to produce dispersal probability grids before the simulation begins.

#### Basal Area

The basal area of each size-class *j* in cell *k* is equal to the number of individuals of both species (*x* = 1 or 2) of that size-class times the basal area of size-class *j*:

$$\;BA_{\;k,j}\;=\;BAJJ_{j}*\overset{5}{\underset{j\;=1}{\sum^{\;}}}\overset{2}{\underset{x\;=\;1}{\sum^{\;}}}N_{x,k,j}$$

The “competitive basal area” (*BA*.*comp*) for each size-class *j* in cell *k* is equal to the basal area of all equal or larger size classes.

#### Germination

The germination probability for seeds of genotype *i* in cell *k* depends on the maximum germination rate (*max*.*germ*), how far the genotype is from its optimum climate (*step*_*i*,*k*_) and the total basal area of the cell.

$$germ_{i,k}\;=\;max.germ\text{ *}\left(1-\left[\left(0.15\text{ * }step_{i,k}\right)+\left(0.069\text{ *}\overset{5}{\underset{j\;=\;1}{\sum^{\;}}}\;BA_{k,j}\right)\right]\right)$$

The total number of new seedlings resulting from germination is the germination probability multiplied by the number of seeds of genotype *i*, rounded to the nearest whole number.

#### Survival

The survival probability for seeds of genotype *i* of size-class *j* in cell *k* depends on the maximum survival of that size-class (*max*.*surv*_*j*_), how far the genotype is from its optimum climate (*step*_*i*,*k*_), and the basal area of equal or larger size-classes. There are 6 genotypes, 3 for each species.

$$surv_{i,j,k}\;=\;max.\;surv_{j}\text{* }\left(1-\left[\left(Sclim_{j}\text{ * }step_{i,k}\right)\;+\;\left(BA.\;eff_{j}\text{ * }Comp.BA_{j,k}\right)\right]\right)$$

The number of individuals in each genotype-size-class in each cell surviving is drawn from a binomial distribution with parameter *surv*_*i*,*j*,*k*_.

#### Pollen dispersal

The number of pollen grains produced by each genotype *i* of species *x* in each cell *k* depends on the maximum pollen production of each size class (*max*.*pol*_*j*_), the number of individuals of each size class, and how far the genotype is from its optimum climate (*step*_*i*,*k*_).

$$pol.\;num_{x,i,k}\;=\;\overset{5}{\underset{j\;=\;1}{\sum^{\;}}}\left[\left(max.\;pol_{j}\text{ * }\left[1\;-\;\left(0.15\text{ * }step_{i,k}\right)\right]\right)\text{ * }N_{x,k,j}\right]$$

A tree of genotype CC will produce only C pollen and a tree of genotype WW will produce only W pollen, but a tree of genotype CW will produce 50% C and 50% W pollen. The amount of pollen of each haploid genotype dispersed from cell *k* to cell *k’* is calculated based on the total amount of pollen produced in cell *k* multiplied by the probability that pollen will travel from cell *k* to cell *k’*. Once all the pollen is dispersed, the proportion of pollen received by each cell *k’* carrying the W allele is calculated.

#### Seed genotype determination

The number of ovules produced by each genotype *i* of species *x* in each cell *k* depends on the maximum seed production of each size class (*max*.*fec*_*j*_), the number of individuals of each size class, and how far the genotype is from its optimum climate (*step*_*i*,*k*_).

$$ov.\;num_{x,i,k}\;=\;\overset{5}{\underset{j\;=\;1}{\sum^{\;}}}\left[\left(max.\;fec_{j}\text{ * }\left[1\;-\;\left(0.15\text{ * }step_{i,k}\right)\right]\right)\text{ * }N_{x,k,j}\right]$$

A tree of genotype CC will produce only C ovules and a tree of genotype WW will produce only W ovules, but a tree of genotype CW will produce 50% C and 50% W ovules. The probability that an ovule will be pollinated by W pollen is equal to the proportion of pollen received by the cell that carries this allele. The genotypic ratio of fertilized seed is calculated as:

$${\text{P}}_{\text{ww,sd}}{\text{ = P}}_{\text{w,pol}}{\text{* P}}_{\text{w,ov}}{\text{ P}}_{\text{cw,sd}}\text{ = }\left({\text{P}}_{\text{w,pol}}{\text{* P}}_{\text{c,ov}}{\text{+ P}}_{\text{c,pol}}{\text{* P}}_{\text{w,ov}}\right){\text{ P}}_{\text{cc,sd}}{\text{ = P}}_{\text{c,pol}}{\text{* P}}_{\text{c,ov}}$$

where P_xy,sd_ is the proportion of seeds with genotype XY, P_x_,pol is the proportion of arriving pollen carrying allele X, and P_y,ov_ is the proportion ovules produced in the cell carrying allele Y.

#### Seed dispersal

Once the frequency of each seed genotype produced within the cell is calculated, the seeds are dispersed. The number of seeds of each diploid genotype dispersed from cell *k* to cell *k’* is calculated based on the total amount of seed produced in cell *k* multiplied by the probability that seed will travel from cell *k* to cell *k’*.

#### Transition

The probability that an individual of size-class *j* will be counted in size-class *j+1* next year depends on the maximum probability of transition (*max*.*trans*_*j*_), how far the genotype is from its optimum climate, and the basal area of equal or larger size-classes.

$$trans_{i,j,k}\;=\;max.\;trans_{j}\;*\;\left(1\;-\;\left[\left(T\;c{\text{lim}}_{j}\;*\;step_{i,k}\right)\;+\;\left(BA.\;eff_{j}\;*\;Comp.\;BA_{j,k}\right)\right]\right)$$

The number of individuals in each genotype-size-class in each cell that actually transition is drawn from a binomial distribution with parameter *surv*_*i*,*j*,*k*_. This number is subtracted from the number of size-class *j* and added to the number of size-class *j+1*.

#### Disturbance

Some model variants include disturbance. The probability that a given cell will experience a disturbance in a particular time step is equal to 1/FR, where FR is the average fire return interval: 15 years or 25 years. If a disturbance occurs, the number of individuals in each size-class in each cell surviving is drawn from a binomial distribution with the survival parameter for that size class for the appropriate disturbance type (Table 1).

## Results

Differences between model variants were more pronounced after 300 years in the new climate than immediately after the 80 years of climate change (S1 Table). At 80 years, both species tend to have few cells with intermediate frequencies of the W allele (that is, cells dominated by WC adults). This occurs because the cells originally dominated by WC individuals have experienced shifting selective pressures favoring WW offspring (produced in 25% of WC matings) over CC or WC offspring, whereas cells that were originally dominated by CC individuals initially have only a few WC offspring, all due to relatively rare gene flow from lower elevations. In the following 300 years, the area occupied by WC individuals re-expands as seed or pollen carrying W alleles disperse into areas formerly dominated by CC individuals. Variation between runs of a given model variant tended to be low, though variation was higher in scenarios including disturbances due to their more stochastic nature (full results in S1 Table). The results that follow come from 300 years post climate shift. After the climate stabilizes, it takes over 500 years for the species to fully reach the new equilibrium, with the exact length of time depending on the model variant (S5 File). The baseline model takes between 1000 and 1500 years

### Interspecific competition

**Fig 3:**
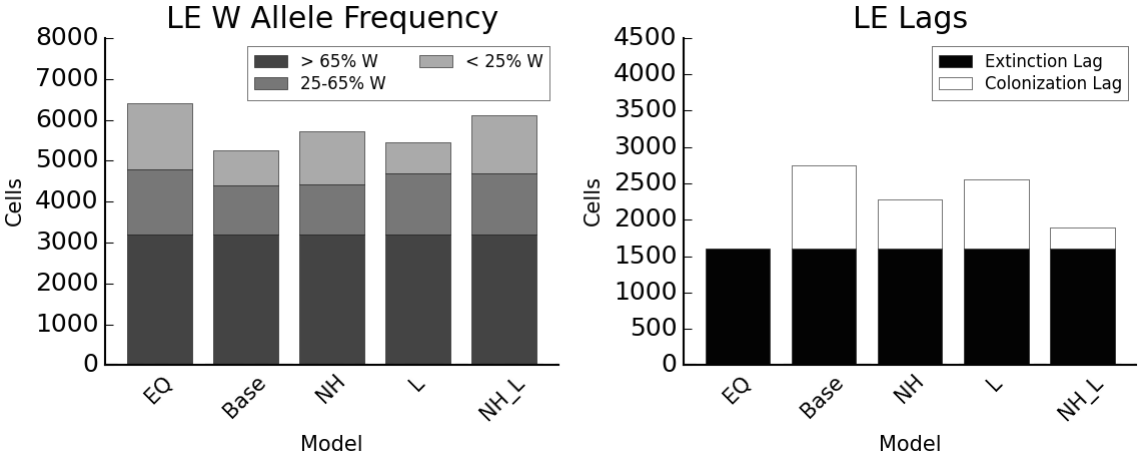
Effects of high-elevation competitor on low-elevation species. EQ: equilibrium distribution after climate change without disturbance; Base: baseline model; NH: no HE species; L: long dispersal; NH_L: no HE species, long dispersal. Y axis indicates total number of cells occupied, colors the genotype category based on proportion of W alleles.

There is significantly less colonization lag in the LE species in the absence of the HE competitor (674.7 suitable cells unoccupied vs. 1146.2; two-sample t-test p = 8.46e^-25^), especially when LE dispersal ability is high (289.5 suitable cells unoccupied vs. 952.1; two-sample t-test p = 5.18e^-28^) (Fig 3). LE reaches its equilibrium distribution approximately 500 years sooner than in the baseline model when HE is absent, even though the area occupied by LE at equilibrium is 25% larger because LE can occupy climates 3–7 in the absence of competition (Figure D in S5 File).

### Dispersal distance, fecundity, and the effects of disturbance

**Fig 4:**
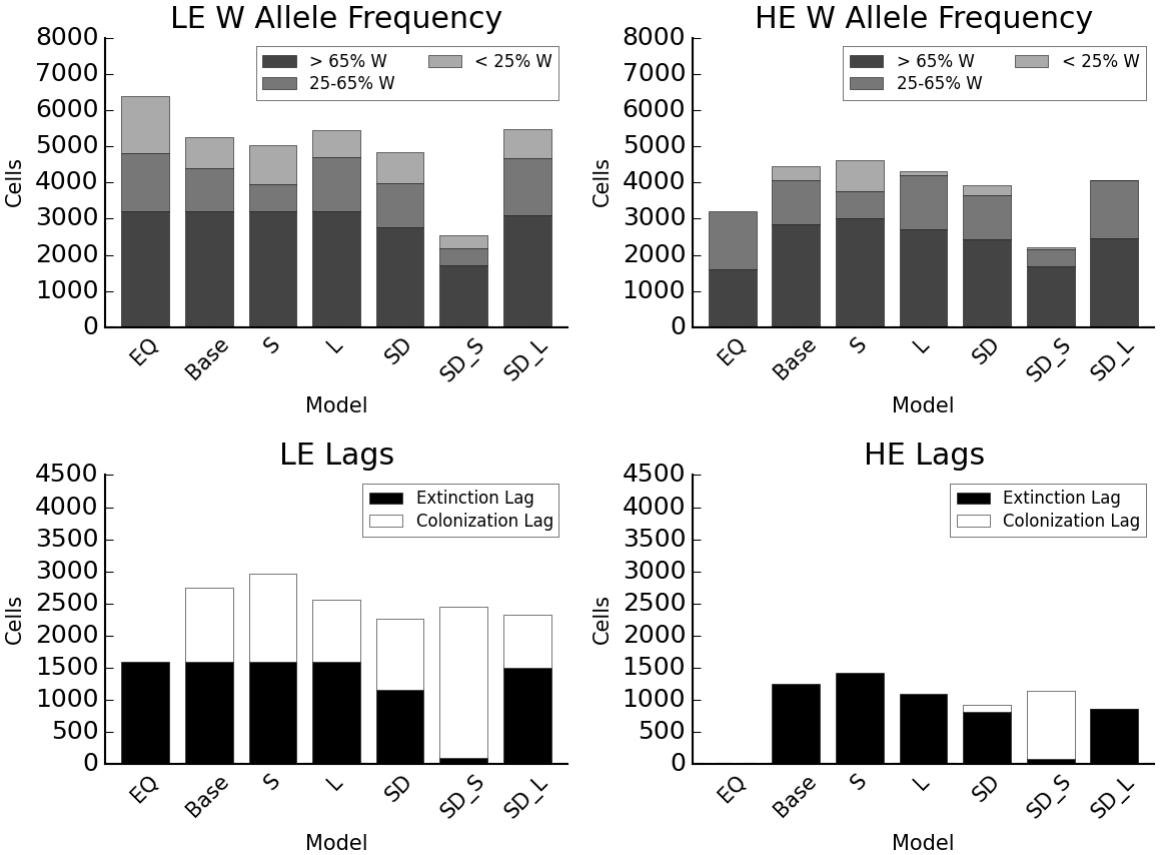
Interacting effects of dispersal distance and disturbance. EQ: equilibrium distribution after climate change without disturbance; Base: baseline model; S: short dispersal; L: long dispersal; SD: simple disturbance; SD_S: SD, short dispersal; SD_L: SD, long dispersal. Y axis indicates total number of cells occupied, colors the genotype category based on proportion of W alleles.

Disturbance tends to reduce extinction lags relative to no-disturbance scenarios, while dispersal distance affects both extinction and colonization lags (Fig 4). Short-distance dispersal increases the range of HE and range of LE in year 380 by about 3.5% relative to the baseline model, while long-distance dispersal decreases HE’s range by 7.5% and increases LE’s range by 3.7%. Simple disturbance reduces the time to equilibrium by about 500 years (Figures A vs. B in S5 File), and at 380 years the area occupied by both species is slightly reduced: by 8.2% in LE due to the presence of disturbed cells and by 12.9% in HE due to the combination of disturbed cells and higher colonization by LE. Dispersal distance interacted with disturbance to affect range dynamics. Short-distance dispersal coupled with simple disturbance reduced range size by about 51% in both species relative to the baseline (Fig 4, column “SD_S” all panels). Long-distance dispersal coupled with simple disturbance reduced range size by 9.7% in HE but increased it by 4.6% in LE (Fig 4, column “SD_L” all panels). Increasing fecundity four times has a similar effect to increasing mean dispersal distance by 50% (Figure A in S6 File). When increased fecundity is combined with reduced dispersal, the higher seed production compensates for the higher-extinction lower-colonization effects of short-distance dispersal (Figure A in S6 File).

**Fig 5:**
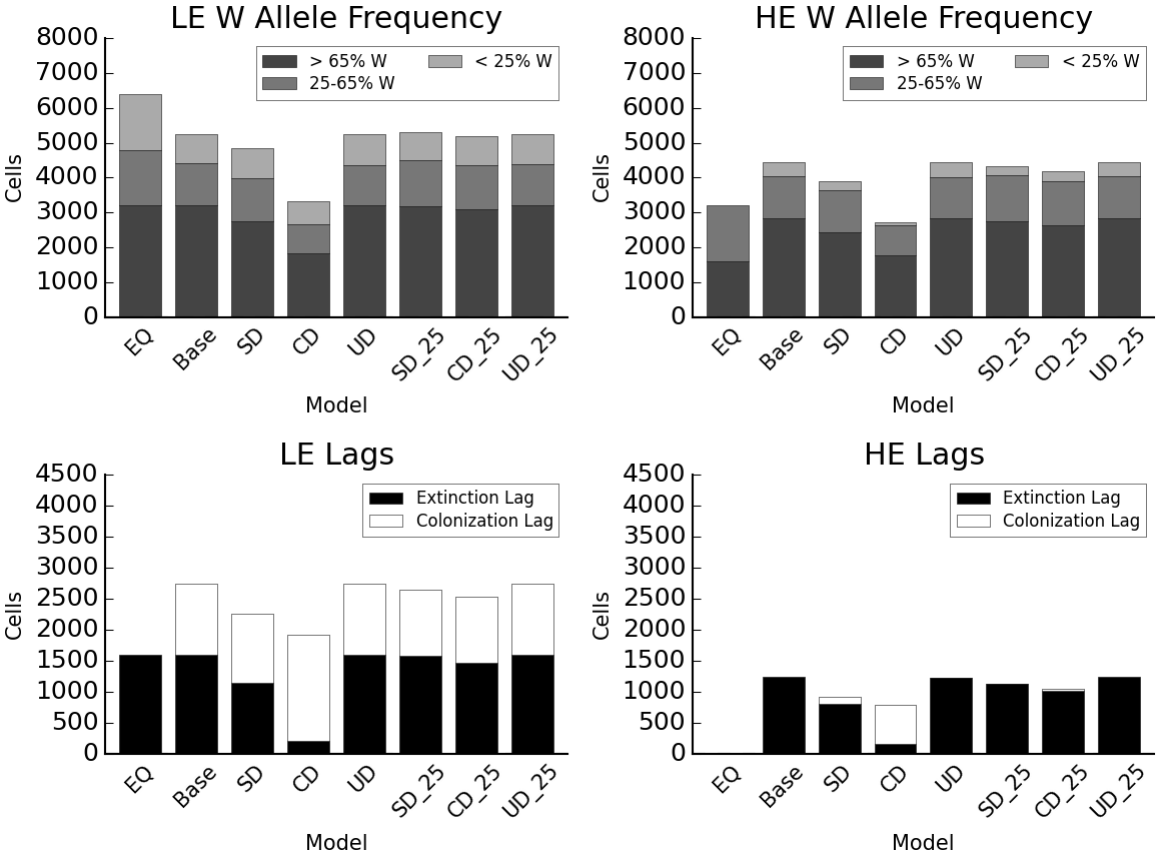
Interaction of disturbance type and frequency. EQ: equilibrium distribution after climate change without disturbance; Base: baseline model; SD: simple disturbance; CD: canopy disturbance; UD: understory disturbance; SD_25: SD, 25 yr. return interval; CD_25: CD, 25 yr. return interval; UD_25: UD, 25 yr. return interval. Y axis indicates total number of cells occupied, colors the genotype category based on proportion of W alleles.

Relative to simple disturbance with a 15-year return interval, equally frequent canopy disturbance leads to higher extinction, lower colonization, and a reduction in range size for both species (38.7% lower in HE, 35.8% in LE, relative to baseline), while understory disturbance leads to less extinction and greater range size (same as baseline in LE, 1% lower in HE) (Fig 5). In both cases, there was an interaction with dispersal distance (Figures B and C in S6 File). Range sizes for HE and LE were 57% and 58.7% lower than baseline when canopy disturbance was combined with short dispersal (29.8% and 35.7% lower than CD alone), but were 2% higher and 5.5% lower than baseline when understory disturbance was combined with short dispersal. Conversely, range sizes for HE and LE were 18.3% and 8.3% lower than baseline when canopy disturbance was combined with long dispersal (33.3% and 42.9% higher than CD alone), but were 4.3% lower and 3.7% higher than baseline when understory disturbance was combined with short dispersal. At longer disturbance intervals (25 years vs. 15), results for the three disturbance types were more similar to each other as well as to the baseline model (Fig 5). At this disturbance interval, HE and LE range sizes relative to the baseline are 3.2% lower and 0.9% higher for simple disturbance, 6.5% lower and 0.9% lower for canopy disturbance, and equal for understory disturbance.

### Genetic variation

**Fig 6:**
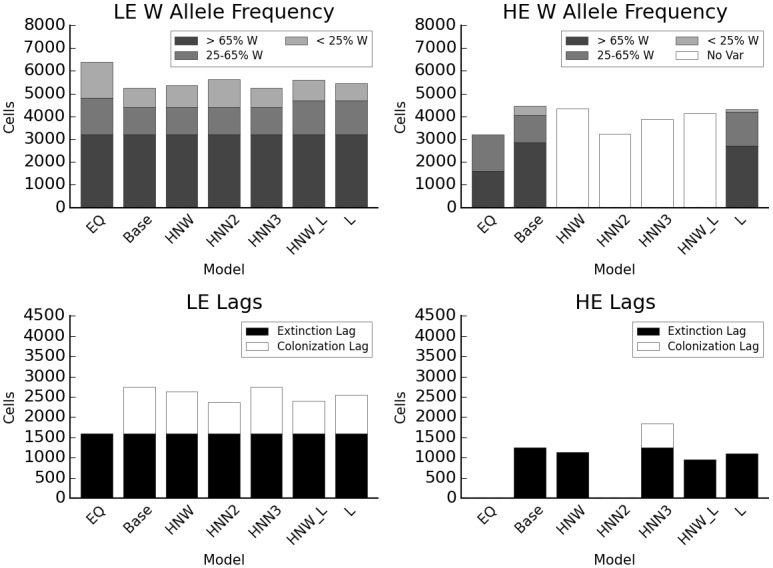
Interaction between genetic diversity and dispersal. EQ: equilibrium distribution after climate change without disturbance; Base: baseline model; HNW: HE species single genotype with wide tolerance (optimum climate 2); HNN2: HE species single genotype with narrow tolerance (optimum 2); HNN3: HE species single genotype with narrow tolerance (optimum 3); HNW_L: HE species single genotype with wide tolerance (optimum 2), long dispersal; L: long dispersal. Y axis indicates total number of cells occupied, colors the genotype category based on proportion of W alleles.

When the HE species is made up of a single genotype with broad environmental tolerances, with an optimum climate of 2, there is less colonization lag in LE and less extinction lag in HE (two-sample t-test p = 2.37e^-11^; p = 1.12e^-12^; Fig 6), indicating that LE is better able to invade the warm range edge of HE, where the HE competitor has lower fitness than in the baseline model (Fig 2). The time to equilibrium in this scenario is similar to the scenario with no high-elevation competitor, but LE cannot occupy climate 3 (Figure C in S5 File). This effect is stronger with long dispersal distances (range occupied by HE and LE in year 380 is 3.2% lower and 1.8% higher in HNW compared to baseline, but 7.5% lower and 6.4% higher in HNW_L), but depends on HE’s optimum climate. In the narrow tolerance scenarios, when HE does best in climate 3 (warm edge), then LE responds similarly to the baseline model, with no increase in colonization, while HE exhibits colonization lag (Fig 6 columns “Base” and “HNN_2”). If the narrow-tolerance HE’s single genotype performs optimally in climate 2, then LE shows even less colonization lag than in the broad-tolerance model and occupies 6.4% more area than in the baseline model, while HE shows greatly reduced range size (28% lower) due to high extinction (Fig 6). If the LE species has a single genotype with broad tolerances and an optimum climate of 5, it exhibits increased lags in colonization (but an equivalent range size to the baseline model) while HE exhibits a 28% greater range size than in the baseline model (Figure D in S6 File). If the HE species has a single genotype with broad tolerance, in the presence of simple disturbance, range sizes in year 380 are 26.9% lower for HE but only 8.3% lower for LE (Figure D in S6 File).

## Discussion

The lags in response observed in these simulations are substantial, with the new equilibrium state not being fully reached until more than 1000 years after the climate stabilizes (S5 File), even though the period of climate change was just 80 years and the equilibrium range boundary moved by only 4 km. This corresponds to a movement of species and genotypes of 2.5–6.9 m/year. Three hundred years after the shift, there were large differences between model variants in the distribution of genotypes, as well as in the amount of newly-suitable habitat unoccupied and less-suitable habitat occupied. The baseline dispersal ability for our simulated species is somewhat optimistic for pine, although our fecundity estimates were rather conservative. Additionally, the simulated adult trees have a relatively high rate of mortality. While we did not track individuals, transition probabilities in the model are such that most surviving seedlings would transition to medium trees within 10–40 years, at which point they reach their maximum survival probability of 0.98/year. With this survival rate, it is unlikely an adult would live more than 300 years, whereas in the wild Jeffrey pines may live for more than 400 years and ponderosa pines for more than 600 [44]. Therefore, the model yields a conservative estimate of the speed of range shifts, as higher adult survival or lower dispersal would only increase the observed extinction and colonization lags. With an increase in maximum large tree survival probability from 0.98 to 0.992 (model variant LTS, in which ~2% of individuals that reach adulthood would survive >500 years), there was a significant increase in both LE colonization lag and HE extinction lag (two-sample t-test p = 3.63e^-8^, p = 3.68e^-11^).

While the high-dispersal scenario is likely overly optimistic, higher fecundity provided a similar boost to the spread of species and genotypes. These elevated fecundity values (16, 240, and 1,600 viable seeds/year for 5–29 cm trees, 30–69 cm trees, and 70–100 cm trees) are not unreasonable for pines if years favorable for seed production are frequent. Reliable dispersal estimates can be difficult to obtain for many species, and the sensitivity of spread rates to both mean dispersal distance and the shape of the tails of the dispersal kernel [5,22,24,53] suggest the need for more accurate, freely available seed and pollen dispersal data. Empirical seed dispersal measurements are challenging in trees, but models can help. Mechanistic models can produce fairly reliable predictions of seed dispersal distances based on seed traits, tree height, and wind conditions [54–57]. Measuring dispersal in animal-dispersed species is more complicated, but there have been some efforts to develop mechanistic models based on animal movement and handling behaviors [58], and genetic marker data can also be used to reveal cryptic dispersal patterns [59–62]. Further progress will require a more general description of disperser behavior (travel distance, etc.) combined with functions linking this behavior to predictive factors (e.g. landscape structure) [63,64].

As one might expect, both the presence of competitors in newly suitable habitat and dispersal limitation tend to restrict the upslope shift of the LE species. When dispersal and/or fecundity are high (low dispersal limitation), cell-scale (50m x 50m) disturbance that removes ~70% of individuals tended to accelerate the shift toward equilibrium conditions (Figures C and D in S6 File), reducing extinction lag and enabling establishment of better-adapted species or genotypes. However, with short distance dispersal, canopy or “simple” disturbance that removed 70% or more of adult trees reduced range size for both species. Without enough individuals at full adult fecundity, there is a decreased chance of seed dispersal to newly suitable habitat; the limited quantity of seed means that there are not enough seedlings to take advantage of the low-competition environments created by canopy disturbance. Reducing disturbance frequency to an average 25-year interval mitigated this effect, but increased extinction lags and did not increase colonization by better-adapted species and genotypes. Understory disturbance did not change range size by more than 4.3% regardless of dispersal distance. In real ecosystems, climate change may increase disturbance frequency by promoting fires or insect outbreaks [30,33,65]. Our results suggest that such disturbance could enable a faster approach to the new equilibrium—but only with high dispersal/fecundity. If dispersal distances combined with fecundity make colonization of the center of large disturbances unlikely or if severe disturbances re-occur on timescales shorter than the average generation times of the species, this could have a negative effect on range size, range shifts, and forest biomass.

Low numbers of individuals near a range edge can slow range shifts by reducing the amount of available seed [66]. In this simulation, greater LE species presence across the range edge in climate 3 before the period of climate shift may have decreased colonization lags by giving LE a head start in areas where it would soon have higher fitness than HE (S4 File). For instance, in scenarios with no genetic variation in HE and an optimum climate of 2, the LE species was able to establish more individuals in climate 3. Conversely, lower LE fitness in climate 4 due to lack of genetic variation decreased population densities in that band, which likely contributed to increased LE colonization lag and decreased HE extinction lag. In this study, even a low-diversity HE species effectively inhibited the range shift of the LE species when it had high fitness near the range border. These results, along with studies showing that dividing species into locally-adapted sub-populations can affect estimates of range shifts under climate change [67–69], suggest that genetic diversity and local adaptation must be taken into account when assessing not only individual species’ vulnerability to climate change, but also the effects of interspecific competition on range shift potential. In future studies, we will investigate how more realistic multi-locus variation and ecological processes may influence range-edge dynamics.

One issue this simulation did not address was heterogeneity, both spatial and temporal. Spatial heterogeneity has the potential to slow population spread when migrants must cross unsuitable habitat [25,70–72]. On the other hand, spatial heterogeneity can create refugia for species within an otherwise less suitable climate that may slow extinction or provide small centers from which populations can expand during periods of environmental change [73,74]. Real forests also experience temporal heterogeneity both in seed production and in the suitability of weather conditions for seedling establishment. If, for instance, suitable conditions for recruitment are rare, then in many years the range edge may remain static, only moving forward when suitable conditions (in terms of weather or disturbance) coincide with a year of high seed production [66,75]. For instance, Nabel et al. found that stochastic variation in climate time sequences had significant effects on the simulated spread of *Ostrya carpinifolia*, and tended to reduce the average spread rate [76]. However, even in these more heterogeneous cases, we would expect that the presence of competitors (and the relative fitness of those competitors), fecundity, dispersal distance, and the nature and frequency of disturbance would affect range shifts in qualitatively similar ways—for instance, that patches of unsuitable habitat would tend to slow spread rates, but that long dispersal or high fecundity would still enable faster spread than short dispersal or low fecundity.

The extent to which the lags in range shifts compared to climate shifts predicted by this and other models will impact forest function and diversity are a topic of much debate. To match shifts in mean temperature predicted under the IPCC 2007 A1B scenario, species would have to move 10–700 m/year upslope in montane areas and 100–10,000 m/year poleward in flatter areas [13]. The A1B scenario was a relatively severe “business as usual” scenario, in which we would expect a 2.9–4.5°C in average global temperature between 2000 and 2100, or 0.029–0.045°C/year on average [77]; a similar rate of change is expected for the RCP 8.5 scenario in the 2013 analysis [78]. The average rate of climate change during de-glaciation 19–11 KYA was 2 orders of magnitude slower than is predicted for the next century, while even the rapid cooling and warming trends in the Younger Dryas 13–12 KYA were one order of magnitude slower [79]. More recent climate shifts 7–4.5 KYA and 6200–165 YA were similarly slower than projected climate shifts due to anthropogenic factors [80]. After the last glacial maximum, tree species in North America and Europe are estimated to have shifted their ranges at around 5–260 m/year, with most species moving <100 m/yr [73,81,82]. Early-successional tree species, which have high fecundity and seed dispersal and short generation times were among the fastest spreading and appear to have quickly moved into the low-competition environments opened up by the retreat of the ice [81,82]. Mid-late successional species lagged behind, as they produce fewer seeds, mature slowly, and would have also been experiencing a more competitive environment [81,82].

If demographic rates calculated under optimal conditions are applicable, the TreeMig model predicted that early successional European tree species should also be able to track future climate change with minor lags, while late successional species may show almost no range shifts [38]. However, even tree species capable of migrating >200 m/year would tend to suffer migration and adaption lags in areas such as eastern North America, central Eurasia, and much of Africa and Australia, where mean temperature isoclines are expected to shift at >1 km/year [13,26]. Moreover, demographic rates, and the factors affecting them, can vary considerably over a species’ range. For instance, if species are not well adapted to range-edge conditions, as simulated in our “low-diversity” scenarios, seed production or growth in these areas could be considerably lower than in the center of the range, impeding range shifts. Many tree species do exhibit local adaptation [3,9], and seed production per tree does not necessarily decline toward the range edge, though low numbers of reproductive individuals can still constrain range spread [3,66]. Some high latitude/elevation populations, either due to gene flow from the range center or lags in adaptation following post-glacial range expansion, are currently maladapted, exhibiting higher growth potential when grown in slightly warmer conditions [83,84]. Growth rate is important for survival in a competitive environment as well as in determining time to reproductive maturity. In such cases, a small amount of climate warming could increase growth—but not if the change continued and outpaced the populations’ adaptive capacity. The effects of climate change on seed production are not entirely clear, but warm springs with late frosts can lead to loss of seed crops in many temperate tree species, while stressful conditions such as drought can lead to cone or fruit abortion [26]. Other environmental changes could also lead to changes in growth and fecundity. For instance, *Pinus taeda* grown under elevated CO_2_ matured at smaller sizes and produced more seed [85]. This would tend to accelerate responses to climate change [57], but it is unknown how common or how strong such responses might be. Changes in wind conditions [57], or availability of animal pollinators and seed dispersers [86] could affect the ability to colonize new areas as well.

Most tree species today, except for those living at the tree line, will need to establish in the presence of competing tree species if they are to shift their ranges. While in our model species only interact at the range edge, in reality trees experience competition from co-occurring species in both central and edge environments. At least one recent study [42] found that the strength of competitive effects on growth was similar across the elevation range of three conifer species. Aitken et al. [3] suggest that reductions in fitness due to lags in climate change responses might tend to weaken competition by reducing the growth of most tree species, which would tend to facilitate persistence in sub-optimal conditions. However, there is certainly no guarantee that species would be equally affected—as mentioned previously, early successional trees may be more likely to be able to migrate and/or adapt to changing climate—and changes in relative fitness could lead to changes in species composition across wide areas.

Disturbance affects the probability of colonization by removing competitors and altering the availability of light, water, and other resources, as well as by altering local temperature and in some cases the level of organic material covering the soil. The effects of different types and frequencies of disturbance on the potential for range shifts in forest trees have not been fully explored. The strong interactions observed in this analysis between disturbance type and frequency and the amount and dispersal distance of seed suggests that this is an issue that deserves more attention. On small scales, forest gap models (e.g. Sortie [87]) can simulate the effects of different types of disturbance on forest structure; at larger landscape scales, it is necessary to aggregate these effects for computational efficiency, as has been done in the TreeMig model [82].

Due to the rapid rate of climate anthropogenic current climate change compared to past climate shifts, the constraints imposed on tree populations by other global change factors such as habitat fragmentation, and the natural constraints on speed of response such as dispersal distance, competition, and time to maturity, there has been increasing interest in the idea of assisted gene flow (AGF) or assisted migration (AM) [11,88–94]. Aitken and Whitlock proposed a framework for assessing the benefits and risks of such assistance based on the level of natural gene flow and the population size of the species: a) the risk of AGF creating outbreeding depression might be important for populations with very low historical gene flow, b) for small populations with moderate to high gene flow, AGF could reduce inbreeding depression or provide demographic support, c) for moderate to large populations with moderate gene flow, AGF might increase the number of alleles that might increase fitness under climate change, but could also introduce maladapted alleles, while d) for large populations with high gene flow AGF would have little effect [88]. Common tree species such as ponderosa pine typically exhibit large effective population sizes, evidence of fairly extensive gene flow at neutral markers [8,95–97]–suggesting that they fall into category c or d—as well as local adaptation [98–101], which suggests natural selection should be fairly effective in purging any maladapted alleles that may be introduced [88]. Rarer tree species would fall into category b, but since trees typically have extensive dispersal compared to non-woody plants relatively few are likely to fall into category a (for which AGF would likely be harmful). It is unclear how many tree species would fall into category d, for which AGF would be superfluous, but some early successional species might qualify. The extent to which tree species or forests might require or benefit from AGF or AGM will depend also on whether there are barriers to dispersal between current and projected future suitable climates [94]; how much migration rates are likely to lag behind climate change given fecundity, dispersal ability, growth rate, establishment requirements, disturbance regime, and the presence of competitors; and the extent to which existing genetic variation will allow adaptation to novel conditions. Trees may be able to persist in areas of sub-optimal climate for long periods if undisturbed, but ecosystem function may be impaired if these species or genotypes are growing slowly and sequestering little carbon, producing few seeds, etc. Our results suggest that some of the species that might benefit from AGF or AGM include species with relatively short dispersal distances and/or small numbers of viable seed (consistent with framework above) especially if they are in an area subject to frequent canopy-disrupting disturbances, as well as those exhibiting low fitness (lack of local adaptation) at the leading edge of their range.

## Conclusion

We found that even under the most optimistic model scenarios there was still a significant lag in the shift of the species’ boundary relative to optimum climate. Our results suggest that interactions between interspecific competition, adaptive potential, and disturbance frequency, together with dispersal ability, are indeed likely to affect tree species’ ranges shift in response to climate change. They also highlight the need for further data on these metrics to understand the nuances of their interactions in specific forest ecosystems, to better predict the response of forests to climate change, and to assess when human intervention (e.g. assisted migration) may be needed to support ecosystem function. Better estimates of fecundity and dispersal, as well as responses to different types of disturbance, are particularly needed.

## Supporting Information

S1 File Baseline model code. Also available on GitHub: https://github.com/emoran5/range_shift_model
(DOCX)

S2 File Parameters and justification. (DOCX)

S3 File Stable size-class distribution for baseline model. (DOCX)

S4 File Occupation of climate 3 by LE and climate 4 by HE prior to climate change for all model variants. (DOCX)

S5 File Approach to stable equilibrium post climate change for baseline model (Figure A), SD (Figure B), NH (Figure C), and HNW (Figure D). (DOCX)

S6 File Figures showing results of additional model variants, including interactions between dispersal and fecundity (Figure A), dispersal and disturbance type (Figures B and C), and the effects of genetic variation (Figure D). (DOCX)

S1 Table Output for all model variants after 80 years of climate change, and after 300 years in new stable climate. (XLSX)
