# Supplementary material for: Simulating the Interacting Effects of Intraspecific Variation, Disturbance, and Competition on Climate-Driven Range Shifts in Trees
Source: PLoS One. 2015 Nov 11;10(11):e0142369. doi: 10.1371/journal.pone.0142369 (PMC4641630; doi:10.1371/journal.pone.0142369)
Supplement: S1 File — Also available on GitHub: https://github.com/emoran5/range_shift_model (DOCX) [file pone.0142369.s001.docx]

**S1: Baseline model, R code**

#20 x 480 50mx50m plot landscape w/6 climates. Species 1 does best in climates 1-3, species 2 in climates 4-6,

# Columns 1-80 climate 6, col 81-160 climate 5, col 161-240 climate 4; col 241-320 climate 3, col 321-400 climate 2, col 401-480 climate 1.

# warm climate genotype (WC) started in WC area, CC in CC area, run for 150 yrs.

# in year 151, WC starts advancing by 1 col every year, so that after 80 years 1/3 of species 1's habitat is gone. Run 300 more years in stable climate.

#Genotype x climate interaction for vital rates - in hotter climate, WC plants have higher establishment, lower mortality, and higher growth

#No disturbance

#####Simulation run time

#Setup initial conditions

TT1 <- 1:150

#Climate change years

TT2 <- 1:80

#New stable conditions

TT3 <- 1:300

##############Setup plot

Plot.width <- 20

Plot.length <- 480

C.width <- Plot.length/6

CellNum <- (Plot.width*Plot.length)

#Habitat cells

KK <- 1:CellNum

#habitat cell quality - climate, given by 1 (cold) to 6 (hot)

kq <- c(rep(6,Plot.width*C.width),rep(5,Plot.width*C.width),rep(4,Plot.width*C.width),rep(3,Plot.width*C.width),rep(2,Plot.width*C.width),rep(1,Plot.width*C.width))

#Cell carrying capacity in basal area - uniform

CCC <- rep(18,length(KK))

#Cell indexing - form into rectangle

index <- cbind(row=rep(1:Plot.length,each=Plot.width),column=rep(1:Plot.width,Plot.length)) #xy coord

clim.state <- matrix(kq,ncol=Plot.width,nrow=Plot.length,byrow=T)

# Makes color-coded image of climate bands image(x=c((1:Plot.length)*50),y=c((1:Plot.width)*50),z=clim.state,col=c('white','beige','yellow','gold','orange1','orange2'),breaks=c(0,1,2,3,4,5,6),xlab="Distance",ylab="Distance",main="climate")

#'white','beige','yellow','gold','orange1','orange2','orange3','red2','red3','red4'

########Tree population

#Size classes - 5 size classes

JJ <- 1:5

#Average basal area for each class

BAJJ <- c(0.0001,0.003,0.07,0.14,0.5)

#Genotypes - 2 homo + 1 heterozygote: cc,cw,ww

II <- 1:3

#Initial population structure

n.entry <- length(KK)*length(II)*length(JJ)

#habitat cells (9600) x genotypes (3) x size classes (5)

N1.i <- rep(0,n.entry)

dim(N1.i) <- c(length(KK),length(JJ),length(II)) #9600 x 5 matrix, set of 3

N2.i <- rep(0,n.entry)

dim(N2.i) <- c(length(KK),length(JJ),length(II)) #9600 x 5 matrix, set of 3

##genotypes of the two species: cc,cw,ww

for(k in KK){

#Climate 1...6

if(kq[k]==6){

N2.i[k,1,] <- c(0,0,5)

N2.i[k,2,] <- c(0,0,20)

N2.i[k,3,] <- c(0,0,18)

N2.i[k,4,] <- c(0,0,6)

N2.i[k,5,] <- c(0,0,2)

}

if(kq[k]==5){

N2.i[k,1,] <- c(0,5,0)

N2.i[k,2,] <- c(0,20,0)

N2.i[k,3,] <- c(0,18,0)

N2.i[k,4,] <- c(0,6,0)

N2.i[k,5,] <- c(0,2,0)

}

if(kq[k]==4){

N2.i[k,1,] <- c(5,0,0)

N2.i[k,2,] <- c(20,0,0)

N2.i[k,3,] <- c(18,0,0)

N2.i[k,4,] <- c(6,0,0)

N2.i[k,5,] <- c(2,0,0)

}

if(kq[k]==3){

N1.i[k,1,] <- c(0,0,5)

N1.i[k,2,] <- c(0,0,20)

N1.i[k,3,] <- c(0,0,18)

N1.i[k,4,] <- c(0,0,6)

N1.i[k,5,] <- c(0,0,2)

}

if(kq[k]==2){

N1.i[k,1,] <- c(0,5,0)

N1.i[k,2,] <- c(0,20,0)

N1.i[k,3,] <- c(0,18,0)

N1.i[k,4,] <- c(0,6,0)

N1.i[k,5,] <- c(0,2,0)

}

if(kq[k]==1){

N1.i[k,1,] <- c(5,0,0)

N1.i[k,2,] <- c(20,0,0)

N1.i[k,3,] <- c(18,0,0)

N1.i[k,4,] <- c(6,0,0)

N1.i[k,5,] <- c(2,0,0)

}

}

##where are the genotypes (adult trees)?

pop.state1 <- rep(0,length(KK));pop.state2 <- rep(0,length(KK))

prop1 <- rep(0,length(KK));prop2 <- rep(0,length(KK))

for(k in KK){

if(sum(N1.i[k,3:5,II])>0){

prop1[k] <- (sum(0.5*N1.i[k,3:5,2])+sum(N1.i[k,3:5,3]))/sum(N1.i[k,3:5,II])

if(prop1[k] <= 0.1){pop.state1[k] <- 1} #<=10% w alleles Sp 1

if(prop1[k] > 0.1 & prop1[k]< 0.3)

{pop.state1[k] <- 2} #10-30% w alleles from Sp 1

if(prop1[k] > 0.3 & prop1[k]< 0.6)

{pop.state1[k] <- 3} #30-60% w alleles from Sp 1

if(prop1[k] >= 0.6){pop.state1[k] <- 4} #>60% w alleles

}

if(sum(N2.i[k,3:5,II])>0){

prop2[k] <- (sum(0.5*N2.i[k,3:5,2])+sum(N2.i[k,3:5,3]))/sum(N2.i[k,3:5,II])

if(prop2[k] <= 0.1){pop.state2[k] <- 1} #<=10% w alleles Sp 2

if(prop2[k] > 0.1 & prop2[k]< 0.3)

{pop.state2[k] <- 2} #10-30% w alleles

if(prop2[k] > 0.3 & prop2[k]< 0.6)

{pop.state2[k] <- 3} #30-60% w alleles

if(prop2[k] >= 0.6){pop.state2[k] <- 4} #>60% w alleles

}

}

mat.pop.state1 <- matrix(pop.state1,ncol=Plot.width,nrow=Plot.length,byrow=T)

prop.mat1 <- matrix(prop1,ncol=Plot.width,nrow=Plot.length,byrow=T)

mat.pop.state2 <- matrix(pop.state2,ncol=Plot.width,nrow=Plot.length,byrow=T)

prop.mat2 <- matrix(prop2,ncol=Plot.width,nrow=Plot.length,byrow=T)

#Color-coded image of allele frequencies

jpeg(filename="InitialPop.jpg",width=6,height=6, units='in', res=500)

par(mfrow=c(2,1))

image(x=c((1:Plot.length)*50),y=c((1:Plot.width)*50),z=mat.pop.state1,col=c("white","yellow","green2","green3","green4"),breaks=c(0,0.5,1.5,2.5,3.5,4.5),xlab="Distance",ylab="Distance",main="Species 1")

legend(800,800,legend=c("Nothing","<10% W allele","10-30% W allele","30-60% W allele",">60% W allele"),fill=c("white","yellow","green2","green3","green4"),cex=0.7)

image(x=c((1:Plot.length)*50),y=c((1:Plot.width)*50),z=mat.pop.state2,col=c("white","yellow","green2","green3","green4"),breaks=c(0,0.5,1.5,2.5,3.5,4.5),xlab="Distance",ylab="Distance",main="Species 2")

#legend(15000,600,legend=c("Nothing","<10% W allele","10-30% W allele","30-60% W allele",">60% W allele"),fill=c("white","yellow","green2","green3","green4"),cex=0.8)

dev.off()

#export as text file

Pop.i <- cbind(N1.i[,,1],N1.i[,,2],N1.i[,,3],N2.i[,,1],N2.i[,,2],N2.i[,,3])

write.table(Pop.i,"InitialPop.txt")

####### Vegetative processes

Ideal.1 <- c(1,2,3); Ideal.2 <- c(4,5,6) #Ideal climate for each genotype (cc,cw,ww)

max.fec <- c(0,0,4,60,400) #maximum seed production for each size class

max.pol <- c(0,0,800,10000,100000) #maximum pollen production for each size class

max.germ <- 0.4 #maximum germination (for now no seed bank)

max.surv <- c(0.6,0.85,0.95,0.98,0.98) #maximum survival for each size class

max.trans <-c(0.15,0.11,0.06,0.02) #maximum transition prob for each size class

Sclim <- c(0.2,0.15,0.08,0.08,0.02) #survival effect of climate

Tclim <- c(0.1,0.08,0.05,0.05) #transition effect of climate

BA.eff <- c(0.06,0.03,0.015,0.01,0) #basal area effect on survival and transition

##########Dispersal

###2D-t seed dispersal

#mean dispersal = 50 m

u <- (50*2/pi)^2

x <- seq(0,400,by=0.1)

pd <- 1/(pi*u*(1+((x^2)/u))^2)

#plot(x,pd,type='l')

sds1 <- rmultinom(1,200000,pd)

sds2 <- numeric(0)

for(s in 1:length(x)){

sds2 <- c(sds2,rep(x[s],sds1[s]))

}

dir <- runif(200000,0,360)

#convert polar to cartesian coordinates

x <- sds2*cos(dir)

y <- sds2*sin(dir)

#plot(x,y)

seed.grid <- matrix(0,17,17) #creates a 17x17 matrix of seed dispersal probabilities, source at (9,9)

xinc <- seq(-425,425,by=50);yinc <- seq(-425,425,by=50);

for(a in 1:length(xinc)){

for(b in 1:length(yinc)){

qq <- length(which(x > xinc[a] & x<= xinc[a+1] & y > yinc[b] & y <= yinc[b+1]))

if(qq>0) seed.grid[a,b] <- qq/length(sds2)

}

}

seed.L <- 17*17

seed.mid <- 145

##2D-t pollen

#mean dispersal = 240 m

u <- (240*2/pi)^2

x <- seq(0,400,by=0.1)

pd <- 1/(pi*u*(1+((x^2)/u))^2)

#plot(x,pd,type='l')

sds1 <- rmultinom(1,200000,pd)

sds2 <- numeric(0)

for(s in 1:length(x)){

sds2 <- c(sds2,rep(x[s],sds1[s]))

}

dir <- runif(200000,0,360)

#convert polar to cartesian coordinates

x <- sds2*cos(dir)

y <- sds2*sin(dir)

#plot(x,y)

pol.grid <- matrix(0,21,21)

xinc <- seq(-525,525,by=50);yinc <- seq(-525,525,by=50);

for(a in 1:length(xinc)){

for(b in 1:length(yinc)){

qq <- length(which(x > xinc[a] & x<= xinc[a+1] & y > yinc[b] & y <= yinc[b+1]))

if(qq>0) pol.grid[a,b] <- qq/length(sds2)

}

}

pol.L <- 21*21

pol.mid <- 221

##Pollen dispersal matrix

PD.mat <- matrix(0,length(KK),length(KK))

for (k in KK){

E <- rep(0,length(KK))

d <- pol.grid

D <- c(d[1,],d[2,],d[3,],d[4,],d[5,],d[6,],d[7,],d[8,],d[9,],d[10,],d[11,],d[12,],d[13,],d[14,],d[15,],d[16,],d[17,],d[18,],d[19,],d[20,],d[21,])

if(k < pol.mid) {

a <- pol.mid - (k-1)

E[1:(k+(pol.mid-1))] <- D[a:pol.L]

}

if(k >= pol.mid & (k+(pol.mid-1)) < length(KK)) E[(k-(pol.mid-1)):(k+(pol.mid-1))] <- D

if((k+(pol.mid-1)) > length(KK)) {

a <- length(KK)-(k+(pol.mid-1))

E[(k-(pol.mid-1)):length(KK)] <- D[1:(pol.L+a)]

}

PD.mat[,k] <- E

}

##Seed dispersal matrix

SD.mat <- matrix(0,length(KK),length(KK))

for (k in KK){

E <- rep(0,length(KK))

d <- seed.grid

D <- c(d[1,],d[2,],d[3,],d[4,],d[5,],d[6,],d[7,],d[8,],d[9,],d[10,],d[11,],d[12,],d[13,],d[14,],d[15,],d[16,],d[17,])

if(k < seed.mid) {

a <- seed.mid - (k-1)

E[1:(k+(seed.mid-1))] <- D[a:seed.L]

}

if(k >= seed.mid & (k+(seed.mid-1)) < length(KK)) E[(k-(seed.mid-1)):(k+(seed.mid-1))] <- D

if((k+(seed.mid-1)) > length(KK)) {

a <- length(KK)-(k-(seed.mid-1))

E[(k-(seed.mid-1)):length(KK)] <- D[1:(a+1)]

}

SD.mat[,k] <- E

}

####MAIN PROGRAM

N1 <- N1.i; N2 <- N2.i #initial population

#Dispersed seeds in the beginning of the simulation - none

sd.cell1 <- matrix(0,length(KK),length(II)) #9600 cells x 3 genotypes

sd.cell2 <- matrix(0,length(KK),length(II))

step1 <- matrix(0,length(KK),3);step2 <- matrix(0,length(KK),3) #How far from ideal?

for(k in KK){

for(i in II){

step1[k,i] <- abs(Ideal.1[i]-kq[k])

step2[k,i] <- abs(Ideal.2[i]-kq[k])

}

}

for(t in TT1){

#Basal area

BA <- matrix(0,length(KK),length(JJ)) #9600 cell x 5 size classes

for(k in 1:length(KK)){ #for each cell, for each size class...

for(j in 1:length(JJ)){

BA[k,j] <- sum(N1[k,j,])*BAJJ[j] + sum(N2[k,j,])*BAJJ[j] #number in size class x average basal area/class

}}

Comp.BA <- matrix(0,length(KK),length(JJ)) #9600 cell x 5 size classes - competitive basal area

#for each size class...

for(j in 1:(length(JJ)-1)){

Comp.BA[,j] <- apply(BA[,j:length(JJ)],1,sum)

}

for(k in KK){

for(i in II){ #germination and survival for each genotype (add competition)

if(t > 1){ #no seeds to germinate, initially

germ1 <- max.germ*(1-((0.15*step1[k,i])+(0.069*sum(BA[k,]))))

germ2 <- max.germ*(1-((0.15*step2[k,i])+(0.069*sum(BA[k,]))))

#Seedling germination

NewSdl1 <- 0; NewSdl2 <- 0

if(germ1 > 0) NewSdl1 <- round(sd.cell1[k,i]*germ1)

if(germ2 > 0) NewSdl2 <- round(sd.cell2[k,i]*germ2)

N1[k,1,i] <- N1[k,1,i]+NewSdl1

N2[k,1,i] <- N2[k,1,i]+NewSdl2

}

#Survival

surv1 <- max.surv*(1-((Sclim *step1[k,i])+(BA.eff*Comp.BA[k,])))

surv2 <- max.surv*(1-((Sclim *step2[k,i])+(BA.eff*Comp.BA[k,])))

for(j in JJ){

if(surv1[j]<0) surv1[j] <- 0; if(surv2[j]<0) surv2[j] <- 0

N1[k,j,i]<- sum(rbinom(N1[k,j,i],1,surv1[j]))

N2[k,j,i]<- sum(rbinom(N2[k,j,i],1,surv2[j]))

}

}

}

#no seed bank from prev. year

sd.cell1 <- matrix(0,length(KK),length(II))

sd.cell2 <- matrix(0,length(KK),length(II))

#Producing seed and pollen

Ov.dist1 <- rep(0,length(KK));Ov.dist2 <- rep(0,length(KK)) #Probability of a "w" ovule in cell K

Pol.dist1 <- rep(0,length(KK));Pol.dist2 <- rep(0,length(KK)) #Probability of a "w" pollen from cell K

Ov.num1 <- rep(0,length(KK));Ov.num2 <- rep(0,length(KK)) #Number of ovules in cell K

Pol.num1 <- rep(0,length(KK));Pol.num2 <- rep(0,length(KK)) #Number of pollen grains cell K

Ov.pres1 <- rep(0,length(KK));Ov.pres2 <- rep(0,length(KK)) #Whether ovules are present

for(k in KK){

#fecundityfor each genotype and stage

fec1 <- matrix(0,3,5); fec2 <- matrix(0,3,5); pol1 <- matrix(0,3,5); pol2 <- matrix(0,3,5)

for(i in II){

if ((0.15*step1[k,i]) < 1) {

fec1[i,] <- max.fec*(1-(0.15*step1[k,i]))

pol1[i,] <- max.pol*(1-(0.15*step1[k,i]))

}

if ((0.15*step2[k,i]) < 1) {

fec2[i,] <- max.fec*(1-(0.15*step2[k,i]))

pol2[i,] <- max.pol*(1-(0.15*step2[k,i]))

}

}

Ov.num1[k] <- sum(N1[k,3:5,1]*fec1[1,3:5])+sum(N1[k,3:5,2]*fec1[2,3:5])+sum(N1[k,3:5,3]*fec1[3,3:5])

Ov.num2[k] <- sum(N2[k,3:5,1]*fec2[1,3:5])+sum(N2[k,3:5,2]*fec2[2,3:5])+sum(N2[k,3:5,3]*fec2[3,3:5])

Pol.num1[k] <- sum(N1[k,3:5,1]*pol1[1,3:5])+sum(N1[k,3:5,2]*pol1[2,3:5])+sum(N1[k,3:5,3]*pol1[3,3:5])

Pol.num2[k] <- sum(N2[k,3:5,1]*pol2[1,3:5])+sum(N2[k,3:5,2]*pol2[2,3:5])+sum(N2[k,3:5,3]*pol2[3,3:5])

if(Ov.num1[k] >0) {

Ov.dist1[k] <- (0.5*sum(N1[k,3:5,2]*fec1[2,3:5])+sum(N1[k,3:5,3]*fec1[3,3:5]))/Ov.num1[k]

Ov.pres1[k] <- 1

}

if(Ov.num2[k] >0) {

Ov.dist2[k] <- (0.5*sum(N2[k,3:5,2]*fec2[2,3:5])+sum(N2[k,3:5,3]*fec2[3,3:5]))/Ov.num2[k]

Ov.pres2[k] <- 1

}

if(Pol.num1[k] >0) Pol.dist1[k] <- (0.5*sum(N1[k,3:5,2]*pol1[2,3:5])+sum(N1[k,3:5,3]*pol1[3,3:5]))/Pol.num1[k]

if(Pol.num2[k] >0) Pol.dist2[k] <- (0.5*sum(N2[k,3:5,2]*pol2[2,3:5])+sum(N2[k,3:5,3]*pol2[3,3:5]))/Pol.num2[k]

}

#Pollen dispersal

#col 1 - total pollen grains received; col 2 - w pollen

Pol.rec1 <- matrix(0,length(KK),2);Pol.rec2 <- matrix(0,length(KK),2)

for (k in KK){

if(sum(N1[k,,])>=1){

temp.pol1t <- Pol.num1[k]*PD.mat[,k]

temp.pol1w <- temp.pol1t*Pol.dist1[k]

Pol.rec1[,1] <- Pol.rec1[,1] + temp.pol1t

Pol.rec1[,2] <- Pol.rec1[,2] + temp.pol1w

}

if(sum(N2[k,,])>=1){

temp.pol2t <- Pol.num2[k]*PD.mat[,k]

temp.pol2w <- temp.pol2t*Pol.dist2[k]

Pol.rec2[,1] <- Pol.rec2[,1] + temp.pol2t

Pol.rec2[,2] <- Pol.rec2[,2] + temp.pol2w

}

}

#proportion of received pollen that is w

Pol.propw1 <- Pol.rec1[,2]/Pol.rec1[,1] ; Pol.propw2 <- Pol.rec2[,2]/Pol.rec2[,1]

Pol.propw1[which(Pol.rec1[,1]==0)] <- 0; Pol.propw2[which(Pol.rec2[,1]==0)] <- 0;

#seed genotype proportions

S.fert1 <- matrix(0,length(KK),3); S.fert2 <- matrix(0,length(KK),3)

S.fert1[,3]<- Ov.dist1*Pol.propw1*Ov.pres1

S.fert1[,2]<- ((Ov.dist1*(1-Pol.propw1) + (1-Ov.dist1)*Pol.propw1))*Ov.pres1

S.fert1[,1]<- (1-Ov.dist1)*(1-Pol.propw1)*Ov.pres1

S.fert2[,3]<- Ov.dist2*Pol.propw2*Ov.pres2

S.fert2[,2]<- ((Ov.dist2*(1-Pol.propw2) + (1-Ov.dist2)*Pol.propw2))*Ov.pres2

S.fert2[,1]<- (1-Ov.dist2)*(1-Pol.propw2)*Ov.pres2

#seed dispersal

for (k in KK){

if(Ov.pres1[k] == 1){

sd.cell1[,1] <- sd.cell1[,1] + (Ov.num1[k]*SD.mat[,k]*S.fert1[k,1])

sd.cell1[,2] <- sd.cell1[,2] + (Ov.num1[k]*SD.mat[,k]*S.fert1[k,2])

sd.cell1[,3] <- sd.cell1[,3] + (Ov.num1[k]*SD.mat[,k]*S.fert1[k,3])

}

if(Ov.pres2[k] == 1){

sd.cell2[,1] <- sd.cell2[,1] + (Ov.num2[k]*SD.mat[,k]*S.fert2[k,1])

sd.cell2[,2] <- sd.cell2[,2] + (Ov.num2[k]*SD.mat[,k]*S.fert2[k,2])

sd.cell2[,3] <- sd.cell2[,3] + (Ov.num2[k]*SD.mat[,k]*S.fert2[k,3])

}

}

sd.cell1 <- round(sd.cell1); sd.cell2 <- round(sd.cell2) #round numbers of seeds

for(k in KK){

for(i in II){ #transition to next size class for each genotype (need to add competition)

trans1 <- max.trans*(1-((Tclim*step1[k,i])+(BA.eff[1:4]*Comp.BA[k,1:4])))

trans2 <- max.trans*(1-((Tclim*step2[k,i])+(BA.eff[1:4]*Comp.BA[k,1:4])))

for(j in 1:4){

if(trans1[j]<0) trans1[j] <- 0; if(trans2[j]<0) trans2[j] <- 0

T1 <- sum(rbinom(N1[k,j,i],1,trans1[j])); T2 <- sum(rbinom(N2[k,j,i],1,trans2[j]))

N1[k,j,i] <- N1[k,j,i]-T1; N1[k,j+1,i] <- N1[k,j+1,i]+T1

N2[k,j,i] <- N2[k,j,i]-T2; N2[k,j+1,i] <- N2[k,j+1,i]+T2

}

}

}

print(t)

} #end setup cycle

#export as text file

Pop.i <- cbind(N1[,,1],N1[,,2],N1[,,3],N2[,,1],N2[,,2],N2[,,3])

write.table(Pop.i,"StablePop1.txt")

##where are the genotypes (adult trees)?

pop.state1 <- rep(0,length(KK));pop.state2 <- rep(0,length(KK))

prop1 <- rep(0,length(KK));prop2 <- rep(0,length(KK))

for(k in KK){

if(sum(N1[k,3:5,II])>0){

prop1[k] <- (sum(0.5*N1[k,3:5,2])+sum(N1[k,3:5,3]))/sum(N1[k,3:5,II])

if(prop1[k] <= 0.1){pop.state1[k] <- 1} #<=10% w alleles Sp 1

if(prop1[k] > 0.1 & prop1[k]< 0.3)

{pop.state1[k] <- 2} #10-30% w alleles from Sp 1

if(prop1[k] > 0.3 & prop1[k]< 0.6)

{pop.state1[k] <- 3} #30-60% w alleles from Sp 1

if(prop1[k] >= 0.6){pop.state1[k] <- 4} #>60% w alleles

}

if(sum(N2[k,3:5,II])>0){

prop2[k] <- (sum(0.5*N2[k,3:5,2])+sum(N2[k,3:5,3]))/sum(N2[k,3:5,II])

if(prop2[k] <= 0.1){pop.state2[k] <- 1} #<=10% w alleles Sp 2

if(prop2[k] > 0.1 & prop2[k]< 0.3)

{pop.state2[k] <- 2} #10-30% w alleles

if(prop2[k] > 0.3 & prop2[k]< 0.6)

{pop.state2[k] <- 3} #30-60% w alleles

if(prop2[k] >= 0.6){pop.state2[k] <- 4} #>60% w alleles

}

}

mat.pop.state1 <- matrix(pop.state1,ncol=Plot.width,nrow=Plot.length,byrow=T)

prop.mat1 <- matrix(prop1,ncol=Plot.width,nrow=Plot.length,byrow=T)

mat.pop.state2 <- matrix(pop.state2,ncol=Plot.width,nrow=Plot.length,byrow=T)

prop.mat2 <- matrix(prop2,ncol=Plot.width,nrow=Plot.length,byrow=T)

jpeg(filename="StablePop1.jpg",width=6,height=6, units='in', res=500)

par(mfrow=c(2,1))

image(x=c((1:Plot.length)*50),y=c((1:Plot.width)*50),z=mat.pop.state1,col=c("white","yellow","green2","green3","green4"),breaks=c(0,0.5,1.5,2.5,3.5,4.5),xlab="Distance",ylab="Distance",main="Species 1")

legend(800,800,legend=c("Nothing","<10% W allele","10-30% W allele","30-60% W allele",">60% W allele"),fill=c("white","yellow","green2","green3","green4"),cex=0.7)

image(x=c((1:Plot.length)*50),y=c((1:Plot.width)*50),z=mat.pop.state2,col=c("white","yellow","green2","green3","green4"),breaks=c(0,0.5,1.5,2.5,3.5,4.5),xlab="Distance",ylab="Distance",main="Species 2")

#legend(15000,600,legend=c("Nothing","<10% W allele","10-30% W allele","30-60% W allele",">60% W allele"),fill=c("white","yellow","green2","green3","green4"),cex=0.8)

dev.off()

####As needed - read in other necessary parameters

bframe1 <-data.frame(read.table("StablePop1.txt",header=T))

#Initial population structure

n.entry <- length(KK)*length(II)*length(JJ)

#habitat cells (9600) x genotypes (3) x size classes (5)

N1 <- rep(0,n.entry)

dim(N1) <- c(length(KK),length(JJ),length(II)) #9600 x 5 matrix, set of 3?

N2<- rep(0,n.entry)

dim(N2) <- c(length(KK),length(JJ),length(II)) #9600 x 5 matrix, set of 3?

N1[,1,1] <- bframe1[,1];N1[,2,1] <- bframe1[,2]; N1[,3,1] <- bframe1[,3];N1[,4,1] <- bframe1[,4];N1[,5,1] <- bframe1[,5]

N1[,1,2] <- bframe1[,6];N1[,2,2] <- bframe1[,7]; N1[,3,2] <- bframe1[,8];N1[,4,2] <- bframe1[,9];N1[,5,2] <- bframe1[,10]

N1[,1,3] <- bframe1[,11];N1[,2,3] <- bframe1[,12]; N1[,3,3] <- bframe1[,13];N1[,4,3] <- bframe1[,14];N1[,5,3] <- bframe1[,15]

N2[,1,1] <- bframe1[,16];N2[,2,1] <- bframe1[,17]; N2[,3,1] <- bframe1[,18];N2[,4,1] <- bframe1[,19];N2[,5,1] <- bframe1[,20]

N2[,1,2] <- bframe1[,21];N2[,2,2] <- bframe1[,22]; N2[,3,2] <- bframe1[,23];N2[,4,2] <- bframe1[,24];N2[,5,2] <- bframe1[,25]

N2[,1,3] <- bframe1[,26];N2[,2,3] <- bframe1[,27]; N2[,3,3] <- bframe1[,28];N2[,4,3] <- bframe1[,29];N2[,5,3] <- bframe1[,30]

####

#habitat cell quality - climate, given by 1 (cold) to 6 (hot)

kq <-c(rep(6,Plot.width*C.width),rep(5,Plot.width*C.width),rep(4,Plot.width*C.width),rep(3,Plot.width*C.width),rep(2,Plot.width*C.width),rep(1,Plot.width*C.width))

for(t in TT2){ #TT2

#climate shift

if(t<=80){

kq <- c(rep(7,Plot.width),kq[1:(CellNum-Plot.width)])

}

if(t>80){

kq <- c(rep(8,Plot.width),kq[1:(CellNum-Plot.width)])

}

#suitability shift

step1 <- matrix(0,length(KK),3);step2 <- matrix(0,length(KK),3) #How far from ideal?

for(k in KK){

for(i in II){

step1[k,i] <- abs(Ideal.1[i]-kq[k])

step2[k,i] <- abs(Ideal.2[i]-kq[k])

}

}

#Basal area

BA <- matrix(0,length(KK),length(JJ)) #9600 cell x 5 size classes

for(k in 1:length(KK)){ #for each cell, for each size class...

for(j in 1:length(JJ)){

BA[k,j] <- sum(N1[k,j,])*BAJJ[j] + sum(N2[k,j,])*BAJJ[j] #number in size class x average basal area/class

}}

Comp.BA <- matrix(0,length(KK),length(JJ)) #9600 cell x 5 size classes - competitive basal area

#for each size class...

for(j in 1:(length(JJ)-1)){

Comp.BA[,j] <- apply(BA[,j:length(JJ)],1,sum)

}

for(k in KK){

for(i in II){ #germination and survival for each genotype (add competition)

germ1 <- max.germ*(1-((0.15*step1[k,i])+(0.069*sum(BA[k,]))))

germ2 <- max.germ*(1-((0.15*step2[k,i])+(0.069*sum(BA[k,]))))

#Seedling germination

NewSdl1 <- 0; NewSdl2 <- 0

if(germ1 > 0) NewSdl1 <- round(sd.cell1[k,i]*germ1)

if(germ2 > 0) NewSdl2 <- round(sd.cell2[k,i]*germ2)

N1[k,1,i] <- N1[k,1,i]+NewSdl1

N2[k,1,i] <- N2[k,1,i]+NewSdl2

#Survival

surv1 <- max.surv*(1-((Sclim *step1[k,i])+(BA.eff*Comp.BA[k,])))

surv2 <- max.surv*(1-((Sclim *step2[k,i])+(BA.eff*Comp.BA[k,])))

for(j in JJ){

if(surv1[j]<0) surv1[j] <- 0; if(surv2[j]<0) surv2[j] <- 0

N1[k,j,i]<- sum(rbinom(N1[k,j,i],1,surv1[j]))

N2[k,j,i]<- sum(rbinom(N2[k,j,i],1,surv2[j]))

}

}

}

#no seed bank from prev. year

sd.cell1 <- matrix(0,length(KK),length(II))

sd.cell2 <- matrix(0,length(KK),length(II))

#Producing seed and pollen

Ov.dist1 <- rep(0,length(KK));Ov.dist2 <- rep(0,length(KK)) #Probability of a "w" ovule in cell K

Pol.dist1 <- rep(0,length(KK));Pol.dist2 <- rep(0,length(KK)) #Probability of a "w" pollen from cell K

Ov.num1 <- rep(0,length(KK));Ov.num2 <- rep(0,length(KK)) #Number of ovules in cell K

Pol.num1 <- rep(0,length(KK));Pol.num2 <- rep(0,length(KK)) #Number of pollen grains cell K

Ov.pres1 <- rep(0,length(KK));Ov.pres2 <- rep(0,length(KK)) #Whether ovules are present

for(k in KK){

#fecundityfor each genotype and stage

fec1 <- matrix(0,3,5); fec2 <- matrix(0,3,5); pol1 <- matrix(0,3,5); pol2 <- matrix(0,3,5)

for(i in II){

if ((0.15*step1[k,i]) < 1) {

fec1[i,] <- max.fec*(1-(0.15*step1[k,i]))

pol1[i,] <- max.pol*(1-(0.15*step1[k,i]))

}

if ((0.15*step2[k,i]) < 1) {

fec2[i,] <- max.fec*(1-(0.15*step2[k,i]))

pol2[i,] <- max.pol*(1-(0.15*step2[k,i]))

}

}

Ov.num1[k] <- sum(N1[k,3:5,1]*fec1[1,3:5])+sum(N1[k,3:5,2]*fec1[2,3:5])+sum(N1[k,3:5,3]*fec1[3,3:5])

Ov.num2[k] <- sum(N2[k,3:5,1]*fec2[1,3:5])+sum(N2[k,3:5,2]*fec2[2,3:5])+sum(N2[k,3:5,3]*fec2[3,3:5])

Pol.num1[k] <- sum(N1[k,3:5,1]*pol1[1,3:5])+sum(N1[k,3:5,2]*pol1[2,3:5])+sum(N1[k,3:5,3]*pol1[3,3:5])

Pol.num2[k] <- sum(N2[k,3:5,1]*pol2[1,3:5])+sum(N2[k,3:5,2]*pol2[2,3:5])+sum(N2[k,3:5,3]*pol2[3,3:5])

if(Ov.num1[k] >0) {

Ov.dist1[k] <- (0.5*sum(N1[k,3:5,2]*fec1[2,3:5])+sum(N1[k,3:5,3]*fec1[3,3:5]))/Ov.num1[k]

Ov.pres1[k] <- 1

}

if(Ov.num2[k] >0) {

Ov.dist2[k] <- (0.5*sum(N2[k,3:5,2]*fec2[2,3:5])+sum(N2[k,3:5,3]*fec2[3,3:5]))/Ov.num2[k]

Ov.pres2[k] <- 1

}

if(Pol.num1[k] >0) Pol.dist1[k] <- (0.5*sum(N1[k,3:5,2]*pol1[2,3:5])+sum(N1[k,3:5,3]*pol1[3,3:5]))/Pol.num1[k]

if(Pol.num2[k] >0) Pol.dist2[k] <- (0.5*sum(N2[k,3:5,2]*pol2[2,3:5])+sum(N2[k,3:5,3]*pol2[3,3:5]))/Pol.num2[k]

}

#Pollen dispersal

#col 1 - total pollen grains received; col 2 - w pollen

Pol.rec1 <- matrix(0,length(KK),2);Pol.rec2 <- matrix(0,length(KK),2)

for (k in KK){

if(sum(N1[k,,])>=1){

temp.pol1t <- Pol.num1[k]*PD.mat[,k]

temp.pol1w <- temp.pol1t*Pol.dist1[k]

Pol.rec1[,1] <- Pol.rec1[,1] + temp.pol1t

Pol.rec1[,2] <- Pol.rec1[,2] + temp.pol1w

}

if(sum(N2[k,,])>=1){

temp.pol2t <- Pol.num2[k]*PD.mat[,k]

temp.pol2w <- temp.pol2t*Pol.dist2[k]

Pol.rec2[,1] <- Pol.rec2[,1] + temp.pol2t

Pol.rec2[,2] <- Pol.rec2[,2] + temp.pol2w

}

}

#proportion of received pollen that is w

Pol.propw1 <- Pol.rec1[,2]/Pol.rec1[,1] ; Pol.propw2 <- Pol.rec2[,2]/Pol.rec2[,1]

Pol.propw1[which(Pol.rec1[,1]==0)] <- 0; Pol.propw2[which(Pol.rec2[,1]==0)] <- 0;

#seed genotype proportions

S.fert1 <- matrix(0,length(KK),3); S.fert2 <- matrix(0,length(KK),3)

S.fert1[,3]<- Ov.dist1*Pol.propw1*Ov.pres1

S.fert1[,2]<- ((Ov.dist1*(1-Pol.propw1) + (1-Ov.dist1)*Pol.propw1))*Ov.pres1

S.fert1[,1]<- (1-Ov.dist1)*(1-Pol.propw1)*Ov.pres1

S.fert2[,3]<- Ov.dist2*Pol.propw2*Ov.pres2

S.fert2[,2]<- ((Ov.dist2*(1-Pol.propw2) + (1-Ov.dist2)*Pol.propw2))*Ov.pres2

S.fert2[,1]<- (1-Ov.dist2)*(1-Pol.propw2)*Ov.pres2

#seed dispersal

for (k in KK){

if(Ov.pres1[k] == 1){

sd.cell1[,1] <- sd.cell1[,1] + (Ov.num1[k]*SD.mat[,k]*S.fert1[k,1])

sd.cell1[,2] <- sd.cell1[,2] + (Ov.num1[k]*SD.mat[,k]*S.fert1[k,2])

sd.cell1[,3] <- sd.cell1[,3] + (Ov.num1[k]*SD.mat[,k]*S.fert1[k,3])

}

if(Ov.pres2[k] == 1){

sd.cell2[,1] <- sd.cell2[,1] + (Ov.num2[k]*SD.mat[,k]*S.fert2[k,1])

sd.cell2[,2] <- sd.cell2[,2] + (Ov.num2[k]*SD.mat[,k]*S.fert2[k,2])

sd.cell2[,3] <- sd.cell2[,3] + (Ov.num2[k]*SD.mat[,k]*S.fert2[k,3])

}

}

sd.cell1 <- round(sd.cell1); sd.cell2 <- round(sd.cell2) #round numbers of seeds

for(k in KK){

for(i in II){ #transition to next size class for each genotype (need to add competition)

trans1 <- max.trans*(1-((Tclim*step1[k,i])+(BA.eff[1:4]*Comp.BA[k,1:4])))

trans2 <- max.trans*(1-((Tclim*step2[k,i])+(BA.eff[1:4]*Comp.BA[k,1:4])))

for(j in 1:4){

if(trans1[j]<0) trans1[j] <- 0; if(trans2[j]<0) trans2[j] <- 0

T1 <- sum(rbinom(N1[k,j,i],1,trans1[j])); T2 <- sum(rbinom(N2[k,j,i],1,trans2[j]))

N1[k,j,i] <- N1[k,j,i]-T1; N1[k,j+1,i] <- N1[k,j+1,i]+T1

N2[k,j,i] <- N2[k,j,i]-T2; N2[k,j+1,i] <- N2[k,j+1,i]+T2

}

}

}

print(t)

} # end climate shift phase

###export as text file

Pop.i <- cbind(N1[,,1],N1[,,2],N1[,,3],N2[,,1],N2[,,2],N2[,,3])

write.table(Pop.i,"PostShiftPop1.txt")

##where are the genotypes (adult trees)?

pop.state1 <- rep(0,length(KK));pop.state2 <- rep(0,length(KK))

prop1 <- rep(0,length(KK));prop2 <- rep(0,length(KK))

occ1 <- rep(0,length(KK));occ2 <- rep(0,length(KK));

for(k in KK){

if(sum(N1[k,3:5,II])>0){

occ1[k] <- 1

prop1[k] <- (sum(0.5*N1[k,3:5,2])+sum(N1[k,3:5,3]))/sum(N1[k,3:5,II])

if(prop1[k] <= 0.1){pop.state1[k] <- 1} #<=10% w alleles Sp 1

if(prop1[k] > 0.1 & prop1[k]< 0.3)

{pop.state1[k] <- 2} #10-30% w alleles from Sp 1

if(prop1[k] > 0.3 & prop1[k]< 0.6)

{pop.state1[k] <- 3} #30-60% w alleles from Sp 1

if(prop1[k] >= 0.6){pop.state1[k] <- 4} #>60% w alleles

}

if(sum(N2[k,3:5,II])>0){

occ2[k]<-1

prop2[k] <- (sum(0.5*N2[k,3:5,2])+sum(N2[k,3:5,3]))/sum(N2[k,3:5,II])

if(prop2[k] <= 0.1){pop.state2[k] <- 1} #<=10% w alleles Sp 2

if(prop2[k] > 0.1 & prop2[k]< 0.3)

{pop.state2[k] <- 2} #10-30% w alleles

if(prop2[k] > 0.3 & prop2[k]< 0.6)

{pop.state2[k] <- 3} #30-60% w alleles

if(prop2[k] >= 0.6){pop.state2[k] <- 4} #>60% w alleles

}

}

mat.pop.state1 <- matrix(pop.state1,ncol=Plot.width,nrow=Plot.length,byrow=T)

prop.mat1 <- matrix(prop1,ncol=Plot.width,nrow=Plot.length,byrow=T)

mat.pop.state2 <- matrix(pop.state2,ncol=Plot.width,nrow=Plot.length,byrow=T)

prop.mat2 <- matrix(prop2,ncol=Plot.width,nrow=Plot.length,byrow=T)

jpeg(filename="PostShiftPop1.jpg",width=6,height=6, units='in', res=500)

par(mfrow=c(2,1))

image(x=c((1:Plot.length)*50),y=c((1:Plot.width)*50),z=mat.pop.state1,col=c("white","yellow","green2","green3","green4"),breaks=c(0,0.5,1.5,2.5,3.5,4.5),xlab="Distance",ylab="Distance",main="Species 1")

legend(800,800,legend=c("Nothing","<10% W allele","10-30% W allele","30-60% W allele",">60% W allele"),fill=c("white","yellow","green2","green3","green4"),cex=0.7)

image(x=c((1:Plot.length)*50),y=c((1:Plot.width)*50),z=mat.pop.state2,col=c("white","yellow","green2","green3","green4"),breaks=c(0,0.5,1.5,2.5,3.5,4.5),xlab="Distance",ylab="Distance",main="Species 2")

#legend(15000,600,legend=c("Nothing","<10% W allele","10-30% W allele","30-60% W allele",">60% W allele"),fill=c("white","yellow","green2","green3","green4"),cex=0.8)

dev.off() ###Gaps - fewer if all age groups, but still there.

###########final climate

clim.state <- matrix(kq,ncol=Plot.width,nrow=Plot.length,byrow=T)

image(x=c((1:Plot.length)*50),y=c((1:Plot.width)*50),z=clim.state,col=c('white','beige','yellow','gold','orange1','orange2','orange3','red2'),breaks=c(0,1,2,3,4,5,6,7,8),xlab="Distance",ylab="Distance",main="climate")

#'white','beige','yellow','gold','orange1','orange2','orange3','red2','red3','red4'

#####

####compare to starting point

bframe2 <-data.frame(read.table("StablePop1.txt",header=T))

N1.s <- rep(0,n.entry)

dim(N1.s) <- c(length(KK),length(JJ),length(II)) #9600 x 5 matrix, set of 3?

N2.s<- rep(0,n.entry)

dim(N2.s) <- c(length(KK),length(JJ),length(II)) #9600 x 5 matrix, set of 3?

N1.s[,1,1] <- bframe2[,1];N1.s[,2,1] <- bframe2[,2]; N1.s[,3,1] <- bframe2[,3];N1.s[,4,1] <- bframe2[,4];N1.s[,5,1] <- bframe2[,5]

N1.s[,1,2] <- bframe2[,6];N1.s[,2,2] <- bframe2[,7]; N1.s[,3,2] <- bframe2[,8];N1.s[,4,2] <- bframe2[,9];N1.s[,5,2] <- bframe2[,10]

N1.s[,1,3] <- bframe2[,11];N1.s[,2,3] <- bframe2[,12]; N1.s[,3,3] <- bframe2[,13];N1.s[,4,3] <- bframe2[,14];N1.s[,5,3] <- bframe2[,15]

N2.s[,1,1] <- bframe2[,16];N2.s[,2,1] <- bframe2[,17]; N2.s[,3,1] <- bframe2[,18];N2.s[,4,1] <- bframe2[,19];N2.s[,5,1] <- bframe2[,20]

N2.s[,1,2] <- bframe2[,21];N2.s[,2,2] <- bframe2[,22]; N2.s[,3,2] <- bframe2[,23];N2.s[,4,2] <- bframe2[,24];N2.s[,5,2] <- bframe2[,25]

N2.s[,1,3] <- bframe2[,26];N2.s[,2,3] <- bframe2[,27]; N2.s[,3,3] <- bframe2[,28];N2.s[,4,3] <- bframe2[,29];N2.s[,5,3] <- bframe2[,30]

##where are the genotypes (adult trees)?

pop.state1s <- rep(0,length(KK));pop.state2s <- rep(0,length(KK))

prop1s <- rep(0,length(KK));prop2s <- rep(0,length(KK))

occ1.s <- rep(0,length(KK));occ2.s <- rep(0,length(KK));

for(k in KK){

if(sum(N1.s[k,3:5,II])>0){

occ1.s[k] <- 1

prop1s[k] <- (sum(0.5*N1.s[k,3:5,2])+sum(N1.s[k,3:5,3]))/sum(N1.s[k,3:5,II])

if(prop1s[k] <= 0.1){pop.state1s[k] <- 1} #<=10% w alleles Sp 1

if(prop1s[k] > 0.1 & prop1s[k]< 0.3)

{pop.state1s[k] <- 2} #10-30% w alleles from Sp 1

if(prop1s[k] > 0.3 & prop1s[k]< 0.6)

{pop.state1s[k] <- 3} #30-60% w alleles from Sp 1

if(prop1s[k] >= 0.6){pop.state1s[k] <- 4} #>60% w alleles

}

if(sum(N2.s[k,3:5,II])>0){

occ2.s[k]<-1

prop2s[k] <- (sum(0.5*N2.s[k,3:5,2])+sum(N2.s[k,3:5,3]))/sum(N2.s[k,3:5,II])

if(prop2s[k] <= 0.1){pop.state2s[k] <- 1} #<=10% w alleles Sp 2

if(prop2s[k] > 0.1 & prop2s[k]< 0.3)

{pop.state2s[k] <- 2} #10-30% w alleles

if(prop2s[k] > 0.3 & prop2s[k]< 0.6)

{pop.state2s[k] <- 3} #30-60% w alleles

if(prop2s[k] >= 0.6){pop.state2s[k] <- 4} #>60% w alleles

}

}

#####population impacts

range.change1 <- sum(occ1)/sum(occ1.s)

range.change2 <- sum(occ2)/sum(occ2.s)

range.change1

range.change2

ideal.prop1 <- rep(0,length(KK));ideal.prop2 <- rep(0,length(KK))

ideal.loc1 <- rep(0,length(KK));ideal.loc2 <- rep(0,length(KK))

ideal.occ1 <- rep(0,length(KK));ideal.occ2 <- rep(0,length(KK))

for (k in KK){

if(kq[k]==1) {

ideal.loc1[k] <- 1

ideal.occ1[k] <- 1

}

if(kq[k]==2) {

ideal.prop1[k] <- 0.5

ideal.loc1[k] <- 1

ideal.occ1[k] <- 1

}

if(kq[k]==3) {

ideal.prop1[k] <- 1

ideal.loc1[k] <- 1

ideal.occ1[k] <- 1

}

if(kq[k]==4) {

ideal.loc2[k] <- 1

ideal.occ2[k] <- 1

}

if(kq[k]==5) {

ideal.prop2[k] <- 0.5

ideal.loc2[k] <- 1

ideal.occ2[k] <- 1

}

if(kq[k]==6) {

ideal.prop2[k] <- 1

ideal.loc2[k] <- 1

ideal.occ2[k] <- 1

}

}

#occupied but unsuitable

length(which(ideal.loc1==0 & occ1==1))

length(which(ideal.loc2==0 & occ2==1))

#unoccupied but suitable

length(which(ideal.loc1==1 & occ1==0))

length(which(ideal.loc2==1 & occ2==0))

#low w frequency obs within area that should be and is occupied;

length(which(occ1==1 & prop1 <= 0.25 & ideal.occ1==1))

length(which(ideal.prop1 <= 0.25 & ideal.occ1==1))

length(which(occ2==1 & prop2 <= 0.25 & ideal.occ2==1))

length(which(ideal.prop2 <= 0.25 & ideal.occ2==1))

#med w frequency obs within area that should be and is occupied;

length(which(occ1==1 & prop1 > 0.25 & prop1 < 0.65 & ideal.occ1==1))

length(which(ideal.prop1 > 0.25 & ideal.prop1 < 0.65 & ideal.occ1==1))

length(which(occ2==1 & prop2 > 0.25 & prop2 < 0.65 & ideal.occ2==1))

length(which(ideal.prop2 > 0.25 & ideal.prop2 <0.65 & ideal.occ2==1))

#high w frequency obs within area that should be and is occupied;

length(which(occ1==1 & prop1 >= 0.65 & ideal.occ1==1))

length(which(ideal.prop1 >= 0.65 & ideal.occ1==1))

length(which(occ2==1 & prop2 >= 0.65 & ideal.occ2==1))

length(which(ideal.prop2 >= 0.65 & ideal.occ2==1))

####As needed - read in other necessary parameters

bframe3 <-data.frame(read.table("PostShiftPop1.txt",header=T))

#Initial population structure

n.entry <- length(KK)*length(II)*length(JJ)

#habitat cells (9600) x genotypes (3) x size classes (5)

N1 <- rep(0,n.entry)

dim(N1) <- c(length(KK),length(JJ),length(II)) #9600 x 5 matrix, set of 3?

N2<- rep(0,n.entry)

dim(N2) <- c(length(KK),length(JJ),length(II)) #9600 x 5 matrix, set of 3?

N1[,1,1] <- bframe3[,1];N1[,2,1] <- bframe3[,2]; N1[,3,1] <- bframe3[,3];N1[,4,1] <- bframe3[,4];N1[,5,1] <- bframe3[,5]

N1[,1,2] <- bframe3[,6];N1[,2,2] <- bframe3[,7]; N1[,3,2] <- bframe3[,8];N1[,4,2] <- bframe3[,9];N1[,5,2] <- bframe3[,10]

N1[,1,3] <- bframe3[,11];N1[,2,3] <- bframe3[,12]; N1[,3,3] <- bframe3[,13];N1[,4,3] <- bframe3[,14];N1[,5,3] <- bframe3[,15]

N2[,1,1] <- bframe3[,16];N2[,2,1] <- bframe3[,17]; N2[,3,1] <- bframe3[,18];N2[,4,1] <- bframe3[,19];N2[,5,1] <- bframe3[,20]

N2[,1,2] <- bframe3[,21];N2[,2,2] <- bframe3[,22]; N2[,3,2] <- bframe3[,23];N2[,4,2] <- bframe3[,24];N2[,5,2] <- bframe3[,25]

N2[,1,3] <- bframe3[,26];N2[,2,3] <- bframe3[,27]; N2[,3,3] <- bframe3[,28];N2[,4,3] <- bframe3[,29];N2[,5,3] <- bframe3[,30]

for(t in TT3){ #Run another 300 years to stabilize

#Basal area

BA <- matrix(0,length(KK),length(JJ)) #9600 cell x 5 size classes

for(k in 1:length(KK)){ #for each cell, for each size class...

for(j in 1:length(JJ)){

BA[k,j] <- sum(N1[k,j,])*BAJJ[j] + sum(N2[k,j,])*BAJJ[j] #number in size class x average basal area/class

}}

Comp.BA <- matrix(0,length(KK),length(JJ)) #9600 cell x 5 size classes - competitive basal area

#for each size class...

for(j in 1:(length(JJ)-1)){

Comp.BA[,j] <- apply(BA[,j:length(JJ)],1,sum)

}

for(k in KK){

for(i in II){ #germination and survival for each genotype (add competition)

germ1 <- max.germ*(1-((0.15*step1[k,i])+(0.069*sum(BA[k,]))))

germ2 <- max.germ*(1-((0.15*step2[k,i])+(0.069*sum(BA[k,]))))

#Seedling germination

NewSdl1 <- 0; NewSdl2 <- 0

if(germ1 > 0) NewSdl1 <- round(sd.cell1[k,i]*germ1)

if(germ2 > 0) NewSdl2 <- round(sd.cell2[k,i]*germ2)

N1[k,1,i] <- N1[k,1,i]+NewSdl1

N2[k,1,i] <- N2[k,1,i]+NewSdl2

#Survival

surv1 <- max.surv*(1-((Sclim *step1[k,i])+(BA.eff*Comp.BA[k,])))

surv2 <- max.surv*(1-((Sclim *step2[k,i])+(BA.eff*Comp.BA[k,])))

for(j in JJ){

if(surv1[j]<0) surv1[j] <- 0; if(surv2[j]<0) surv2[j] <- 0

N1[k,j,i]<- sum(rbinom(N1[k,j,i],1,surv1[j]))

N2[k,j,i]<- sum(rbinom(N2[k,j,i],1,surv2[j]))

}

}

}

#no seed bank

sd.cell1 <- matrix(0,length(KK),length(II))

sd.cell2 <- matrix(0,length(KK),length(II))

#Producing seed and pollen

Ov.dist1 <- rep(0,length(KK));Ov.dist2 <- rep(0,length(KK)) #Probability of a "w" ovule in cell K

Pol.dist1 <- rep(0,length(KK));Pol.dist2 <- rep(0,length(KK)) #Probability of a "w" pollen from cell K

Ov.num1 <- rep(0,length(KK));Ov.num2 <- rep(0,length(KK)) #Number of ovules in cell K

Pol.num1 <- rep(0,length(KK));Pol.num2 <- rep(0,length(KK)) #Number of pollen grains cell K

Ov.pres1 <- rep(0,length(KK));Ov.pres2 <- rep(0,length(KK)) #Whether ovules are present

for(k in KK){

#fecundityfor each genotype and stage

fec1 <- matrix(0,3,5); fec2 <- matrix(0,3,5); pol1 <- matrix(0,3,5); pol2 <- matrix(0,3,5)

for(i in II){

if ((0.15*step1[k,i]) < 1) {

fec1[i,] <- max.fec*(1-(0.15*step1[k,i]))

pol1[i,] <- max.pol*(1-(0.15*step1[k,i]))

}

if ((0.15*step2[k,i]) < 1) {

fec2[i,] <- max.fec*(1-(0.15*step2[k,i]))

pol2[i,] <- max.pol*(1-(0.15*step2[k,i]))

}

}

Ov.num1[k] <- sum(N1[k,3:5,1]*fec1[1,3:5])+sum(N1[k,3:5,2]*fec1[2,3:5])+sum(N1[k,3:5,3]*fec1[3,3:5])

Ov.num2[k] <- sum(N2[k,3:5,1]*fec2[1,3:5])+sum(N2[k,3:5,2]*fec2[2,3:5])+sum(N2[k,3:5,3]*fec2[3,3:5])

Pol.num1[k] <- sum(N1[k,3:5,1]*pol1[1,3:5])+sum(N1[k,3:5,2]*pol1[2,3:5])+sum(N1[k,3:5,3]*pol1[3,3:5])

Pol.num2[k] <- sum(N2[k,3:5,1]*pol2[1,3:5])+sum(N2[k,3:5,2]*pol2[2,3:5])+sum(N2[k,3:5,3]*pol2[3,3:5])

if(Ov.num1[k] >0) {

Ov.dist1[k] <- (0.5*sum(N1[k,3:5,2]*fec1[2,3:5])+sum(N1[k,3:5,3]*fec1[3,3:5]))/Ov.num1[k]

Ov.pres1[k] <- 1

}

if(Ov.num2[k] >0) {

Ov.dist2[k] <- (0.5*sum(N2[k,3:5,2]*fec2[2,3:5])+sum(N2[k,3:5,3]*fec2[3,3:5]))/Ov.num2[k]

Ov.pres2[k] <- 1

}

if(Pol.num1[k] >0) Pol.dist1[k] <- (0.5*sum(N1[k,3:5,2]*pol1[2,3:5])+sum(N1[k,3:5,3]*pol1[3,3:5]))/Pol.num1[k]

if(Pol.num2[k] >0) Pol.dist2[k] <- (0.5*sum(N2[k,3:5,2]*pol2[2,3:5])+sum(N2[k,3:5,3]*pol2[3,3:5]))/Pol.num2[k]

}

#Pollen dispersal

#col 1 - total pollen grains received; col 2 - w pollen

Pol.rec1 <- matrix(0,length(KK),2);Pol.rec2 <- matrix(0,length(KK),2)

for (k in KK){

if(sum(N1[k,,])>=1){

temp.pol1t <- Pol.num1[k]*PD.mat[,k]

temp.pol1w <- temp.pol1t*Pol.dist1[k]

Pol.rec1[,1] <- Pol.rec1[,1] + temp.pol1t

Pol.rec1[,2] <- Pol.rec1[,2] + temp.pol1w

}

if(sum(N2[k,,])>=1){

temp.pol2t <- Pol.num2[k]*PD.mat[,k]

temp.pol2w <- temp.pol2t*Pol.dist2[k]

Pol.rec2[,1] <- Pol.rec2[,1] + temp.pol2t

Pol.rec2[,2] <- Pol.rec2[,2] + temp.pol2w

}

}

#proportion of received pollen that is w

Pol.propw1 <- Pol.rec1[,2]/Pol.rec1[,1] ; Pol.propw2 <- Pol.rec2[,2]/Pol.rec2[,1]

Pol.propw1[which(Pol.rec1[,1]==0)] <- 0; Pol.propw2[which(Pol.rec2[,1]==0)] <- 0;

#seed genotype proportions

S.fert1 <- matrix(0,length(KK),3); S.fert2 <- matrix(0,length(KK),3)

S.fert1[,3]<- Ov.dist1*Pol.propw1*Ov.pres1

S.fert1[,2]<- ((Ov.dist1*(1-Pol.propw1) + (1-Ov.dist1)*Pol.propw1))*Ov.pres1

S.fert1[,1]<- (1-Ov.dist1)*(1-Pol.propw1)*Ov.pres1

S.fert2[,3]<- Ov.dist2*Pol.propw2*Ov.pres2

S.fert2[,2]<- ((Ov.dist2*(1-Pol.propw2) + (1-Ov.dist2)*Pol.propw2))*Ov.pres2

S.fert2[,1]<- (1-Ov.dist2)*(1-Pol.propw2)*Ov.pres2

#seed dispersal

for (k in KK){

if(Ov.pres1[k] == 1){

sd.cell1[,1] <- sd.cell1[,1] + (Ov.num1[k]*SD.mat[,k]*S.fert1[k,1])

sd.cell1[,2] <- sd.cell1[,2] + (Ov.num1[k]*SD.mat[,k]*S.fert1[k,2])

sd.cell1[,3] <- sd.cell1[,3] + (Ov.num1[k]*SD.mat[,k]*S.fert1[k,3])

}

if(Ov.pres2[k] == 1){

sd.cell2[,1] <- sd.cell2[,1] + (Ov.num2[k]*SD.mat[,k]*S.fert2[k,1])

sd.cell2[,2] <- sd.cell2[,2] + (Ov.num2[k]*SD.mat[,k]*S.fert2[k,2])

sd.cell2[,3] <- sd.cell2[,3] + (Ov.num2[k]*SD.mat[,k]*S.fert2[k,3])

}

}

sd.cell1 <- round(sd.cell1); sd.cell2 <- round(sd.cell2) #round numbers of seeds

for(k in KK){

for(i in II){ #transition to next size class for each genotype (need to add competition)

trans1 <- max.trans*(1-((Tclim*step1[k,i])+(BA.eff[1:4]*Comp.BA[k,1:4])))

trans2 <- max.trans*(1-((Tclim*step2[k,i])+(BA.eff[1:4]*Comp.BA[k,1:4])))

for(j in 1:4){

if(trans1[j]<0) trans1[j] <- 0; if(trans2[j]<0) trans2[j] <- 0

T1 <- sum(rbinom(N1[k,j,i],1,trans1[j])); T2 <- sum(rbinom(N2[k,j,i],1,trans2[j]))

N1[k,j,i] <- N1[k,j,i]-T1; N1[k,j+1,i] <- N1[k,j+1,i]+T1

N2[k,j,i] <- N2[k,j,i]-T2; N2[k,j+1,i] <- N2[k,j+1,i]+T2

}

}

}

print(t)

} # end climate shift phase

###export as text file

Pop.i <- cbind(N1[,,1],N1[,,2],N1[,,3],N2[,,1],N2[,,2],N2[,,3])

write.table(Pop.i,"PostShiftStablePop1.txt")

##where are the genotypes (adult trees)?

pop.state1 <- rep(0,length(KK));pop.state2 <- rep(0,length(KK))

prop1 <- rep(0,length(KK));prop2 <- rep(0,length(KK))

occ1 <- rep(0,length(KK));occ2 <- rep(0,length(KK));

for(k in KK){

if(sum(N1[k,3:5,II])>0){

occ1[k] <- 1

prop1[k] <- (sum(0.5*N1[k,3:5,2])+sum(N1[k,3:5,3]))/sum(N1[k,3:5,II])

if(prop1[k] <= 0.1){pop.state1[k] <- 1} #<=10% w alleles Sp 1

if(prop1[k] > 0.1 & prop1[k]< 0.3)

{pop.state1[k] <- 2} #10-30% w alleles from Sp 1

if(prop1[k] > 0.3 & prop1[k]< 0.6)

{pop.state1[k] <- 3} #30-60% w alleles from Sp 1

if(prop1[k] >= 0.6){pop.state1[k] <- 4} #>60% w alleles

}

if(sum(N2[k,3:5,II])>0){

occ2[k]<-1

prop2[k] <- (sum(0.5*N2[k,3:5,2])+sum(N2[k,3:5,3]))/sum(N2[k,3:5,II])

if(prop2[k] <= 0.1){pop.state2[k] <- 1} #<=10% w alleles Sp 2

if(prop2[k] > 0.1 & prop2[k]< 0.3)

{pop.state2[k] <- 2} #10-30% w alleles

if(prop2[k] > 0.3 & prop2[k]< 0.6)

{pop.state2[k] <- 3} #30-60% w alleles

if(prop2[k] >= 0.6){pop.state2[k] <- 4} #>60% w alleles

}

}

mat.pop.state1 <- matrix(pop.state1,ncol=Plot.width,nrow=Plot.length,byrow=T)

prop.mat1 <- matrix(prop1,ncol=Plot.width,nrow=Plot.length,byrow=T)

mat.pop.state2 <- matrix(pop.state2,ncol=Plot.width,nrow=Plot.length,byrow=T)

prop.mat2 <- matrix(prop2,ncol=Plot.width,nrow=Plot.length,byrow=T)

jpeg(filename="PostStableShiftPop1.jpg",width=6,height=6, units='in', res=500)

par(mfrow=c(2,1))

image(x=c((1:Plot.length)*50),y=c((1:Plot.width)*50),z=mat.pop.state1,col=c("white","yellow","green2","green3","green4"),breaks=c(0,0.5,1.5,2.5,3.5,4.5),xlab="Distance",ylab="Distance",main="Species 1")

legend(800,800,legend=c("Nothing","<10% W allele","10-30% W allele","30-60% W allele",">60% W allele"),fill=c("white","yellow","green2","green3","green4"),cex=0.7)

image(x=c((1:Plot.length)*50),y=c((1:Plot.width)*50),z=mat.pop.state2,col=c("white","yellow","green2","green3","green4"),breaks=c(0,0.5,1.5,2.5,3.5,4.5),xlab="Distance",ylab="Distance",main="Species 2")

#legend(15000,600,legend=c("Nothing","<10% W allele","10-30% W allele","30-60% W allele",">60% W allele"),fill=c("white","yellow","green2","green3","green4"),cex=0.8)

dev.off() ###Gaps - fewer if all age groups, but still there.

range.change1 <- sum(occ1)/sum(occ1.s)

range.change2 <- sum(occ2)/sum(occ2.s)

range.change1

range.change2

ideal.prop1 <- rep(0,length(KK));ideal.prop2 <- rep(0,length(KK))

ideal.loc1 <- rep(0,length(KK));ideal.loc2 <- rep(0,length(KK))

ideal.occ1 <- rep(0,length(KK));ideal.occ2 <- rep(0,length(KK))

for (k in KK){

if(kq[k]==1) {

ideal.loc1[k] <- 1

ideal.occ1[k] <- 1

}

if(kq[k]==2) {

ideal.prop1[k] <- 0.5

ideal.loc1[k] <- 1

ideal.occ1[k] <- 1

}

if(kq[k]==3) {

ideal.prop1[k] <- 1

ideal.loc1[k] <- 1

ideal.occ1[k] <- 1

}

if(kq[k]==4) {

ideal.loc2[k] <- 1

ideal.occ2[k] <- 1

}

if(kq[k]==5) {

ideal.prop2[k] <- 0.5

ideal.loc2[k] <- 1

ideal.occ2[k] <- 1

}

if(kq[k]==6) {

ideal.prop2[k] <- 1

ideal.loc2[k] <- 1

ideal.occ2[k] <- 1

}

}

#occupied but unsuitable

length(which(ideal.loc1==0 & occ1==1))

length(which(ideal.loc2==0 & occ2==1))

#unoccupied but suitable

length(which(ideal.loc1==1 & occ1==0))

length(which(ideal.loc2==1 & occ2==0))

#low w frequency obs within area that should be and is occupied;

length(which(occ1==1 & prop1 <= 0.25 & ideal.occ1==1))

length(which(ideal.prop1 <= 0.25 & ideal.occ1==1))

length(which(occ2==1 & prop2 <= 0.25 & ideal.occ2==1))

length(which(ideal.prop2 <= 0.25 & ideal.occ2==1))

#med w frequency obs within area that should be and is occupied;

length(which(occ1==1 & prop1 > 0.25 & prop1 < 0.65 & ideal.occ1==1))

length(which(ideal.prop1 > 0.25 & ideal.prop1 < 0.65 & ideal.occ1==1))

length(which(occ2==1 & prop2 > 0.25 & prop2 < 0.65 & ideal.occ2==1))

length(which(ideal.prop2 > 0.25 & ideal.prop2 <0.65 & ideal.occ2==1))

#high w frequency obs within area that should be and is occupied;

length(which(occ1==1 & prop1 >= 0.65 & ideal.occ1==1))

length(which(ideal.prop1 >= 0.65 & ideal.occ1==1))

length(which(occ2==1 & prop2 >= 0.65 & ideal.occ2==1))

length(which(ideal.prop2 >= 0.65 & ideal.occ2==1))
