# Supplementary material for: Simulating the Interacting Effects of Intraspecific Variation, Disturbance, and Competition on Climate-Driven Range Shifts in Trees
Source: PLoS One. 2015 Nov 11;10(11):e0142369. doi: 10.1371/journal.pone.0142369 (PMC4641630; doi:10.1371/journal.pone.0142369)
Supplement: S2 File — (DOCX) [file pone.0142369.s002.docx]

**S2: Parameter values and justification**

**Initial conditions**

We initialized the model with 5 seedlings, 20 saplings, 18 small trees, 6 medium trees, and 2 large trees of the optimum genotype for the climate in each cell. We set these low values so that high adult basal area would not constrain recruitment. After the first 150 years in the initial stable climate, the size distribution stabilizes at an average of 276.7 seedlings, 49.9 saplings, 20.3 small trees, 10.8 medium trees, and 10.1 large trees per cell for the baseline model. This translates to 7.93 m^2^ adult and 8.11 m^2^ total basal area/patch. This is lower than the simulation maximum density of 18 m^2^ (maximum seedling recruitment is 0 at a total basal area of 14.4 m^2^ or greater although size class transitions can still occur), and higher than the 1.73-3.45 m^2^ adult basal area per quarter-ha that is usually the goal of ponderosa pine thinning treatments [42]. The size distribution did not change significantly for the baseline model when it was run for a further 100 or 200 years (MANOVA, Wilks’ Lambda: p = 0.51) (S3).

**Process order**

In Sierra Nevada pines, seeds germinate in the spring, much of the mortality occurs during summer drought, and seed dispersal occurs in the fall. Therefore, in each time-step, the model simulates first seed germination, then mortality, then pollen and seed dispersal, and finally transitions in size class (which determines the number of each individual per class in the next year).

**Cell carrying capacity**

The carrying capacity of each cell was set to 18 m^2^ of basal area. As a result, at carrying capacity a cell containing only large trees would have 36 trees with about 4.7 m between each, while one containing only saplings, would have 6000 saplings spaced at 2.4 saplings/m^2^.

**Germination**

Maximum germination (max.germ) = 0.4

Effect of distance from optimum climate = 0.15 (reduction per climate “step”); 0.08 (LNW, HNW)

Effect of existing vegetation basal area on germination = 0.069 (reduction per m^2^ BA)

Germination declines [39] by 6.9% of the maximum for every 1m^2^ of total basal area, so that if the cell is at 80% of carrying capacity or above there is no seedling establishment. Transitions to larger size classes decline by the same percentage of the maximum. This simulates the effect of shading on a shade-intolerant species. For example, annual survival of small trees at 18m^2^ basal area of same-size-and-larger trees is 0.69 and the transition probability to the next size class is 0.11 as opposed to 0.95 and 0.15 with no competition. In the baseline model, for every climate “step” a genotype moves away from its ideal climate the maximum germination is reduced by 15% of its original maximum. For instance, a HE species CC seed would have a 0.4 chance of germinating in climate 1 and only a 0.1 chance of germinating in climate 6. Negative demographic rates are set to zero. There is assumed to be no persistent seed bank, so any seeds that do not germinated die.

Maximum germination and the effect of basal area on germination are the same in all model variants. The effect of climate variation on germination is lower for the low-genetic-diversity species in model variants LNW and HNW.

**Survival and Growth**

|  | Seedlings | Saplings | Small trees | Medium trees | Large trees |
| --- | --- | --- | --- | --- | --- |
| Basal Area  (BAJJ) | 0.0001 | 0.003 | 0.07 | 0.14 | 0.5 |
| Max Survival  (max.surv) | 0.6 | 0.85 | 0.95 | 0.98 | 0.98 |
| Max Transition  (max.trans) | 0.15 | 0.11 | 0.06 | 0.02 | NA |
| Effect of climate on survival (LNW; HNW)  (Sclim) | 0.2  0.12 | 0.15  0.08 | 0.08  0.05 | 0.08  0.05 | 0.02  0.012 |
| Effect of climate on transition (LNW; HNW)  (Tclim) | 0.1  0.06 | 0.08  0.05 | 0.05  0.026 | 0.05  0.026 | NA |
| Effect of competitor basal area on survival & transition  (BA.eff) | 0.06 | 0.03 | 0.015 | 0.01 | NA |

Maximum survival probability declines linearly with the sum of the basal area of all equal-or-larger size classes – for seedlings by 6%, saplings 3%, small trees 1.5%, and medium trees 1% of the maximum for each m^2^ of basal area in the baseline model. In the baseline model, survival for a genotype declines 20% of maximum for seedlings, 15% for saplings, 8% for small and medium trees, and 2% for large trees with every step away from the ideal climate. The probability of transition to a larger size class declines 10% relative to the maximum for seedlings, 8% for saplings, and 5% for small and medium trees with every step away from the ideal climate. In this simulation the effects of climate and competition are additive: if climate unsuitability would lower survival by 15% and competition would lower it by 20%, then the individual has a total survival probability of 35% less than its original maximum.

Survival and growth parameters are the same in all model variants, except for Sclim and Tclim in variants LNW and HNW. In these scenarios, the effect of moving away from the optimum climate is reduced in the low-genetic-diversity species.

**Seed and Pollen Production**

|  | Seedlings | Saplings | Small trees | Medium trees | Large trees |
| --- | --- | --- | --- | --- | --- |
| Max fecundity  (max.fec) | 0 | 0 | 4  16 (HF) | 60  240 (HF) | 400  1600 (HF) |
| Max pollen  (max.pol) | 0 | 0 | 800 | 10,000 | 100,000 |
| Effect of climate on seed & pollen production |  |  | 0.15  0.08 (LNW; HNW) | 0.15  0.08 (LNW; HNW) | 0.15  0.08 (LNW; HNW) |

The model simulates the dispersal of viable seed, here defined as seeds that have the potential to germinate (ie. not immature or damaged by predators or pathogens). Seed and pollen production decline 15% of maximum with every step away from the ideal climate. In ponderosa pine, trees 70-100 cm DBH can regularly produce over 100 cones/year, trees 50-70 cm produce some cones most years, trees 30-50 cm produce less than 100 cones most years, and trees <30 cm do not regularly produce cones [40]. Ponderosa pine cones contain 31-70 seeds [41], and a tree can produce up to 2,000 filled seeds per tree in good years [42]. However, many seeds succumb to predators or pathogens before they get a chance to germinate. The values chosen for this model, shown in the table above, are meant to reflect an average between low and high seed years, and early mortality due to predation. Many more pollen grains are produced than seeds, and pollen can be produced by much smaller individuals.

In the high-fecundity model variant, trees produce four times as much seed. Seed and pollen production is reduced 15% for every step away from the optimum climate, except for the low-genetic-diversity species in model variants LNW and HNW, in which they are reduced 8% for every step away from the optimum.

**Seed and pollen dispersal**

|  | Baseline | Long dispersal (L) | Short dispersal (S) |
| --- | --- | --- | --- |
| Seed dispersal parameter (u_s_) | 1013.212 | 2279.727 | 253.303 |
| Pollen dispersal parameter (u_s_) | 23344.4 | 52524.9 | 5836.1 |

In the baseline model, the average seed dispersal distance is 50 m, and the average pollen dispersal distance 240 m, which corresponds to the *u* parameters in the table above. Based on the corresponding 2D-t kernel, 77.4% of seeds and 23.4% of pollen grains remain in their home cells, while the rest disperse. Most seeds fall within 200 m of the source, most pollen grains within 400 m. These values are somewhat optimistic, because while ponderosa pine seeds can occasionally travel up to 10 km, most seem to cluster within 25-50 m of their maternal parent [44,45]. While we could not find a good pollen dispersal estimate for *P. ponderosa* or *P. jeffreyi*, *P. sylvestris* was found to have a fat-tailed pollen dispersal kernel with a mean dispersal distance of 135 m [46].

The high dispersal model variant increases mean seed and pollen dispersal distances by 50% to 75 m and 360 m. The low dispersal model variant decreases mean seed and pollen dispersal distances by 50% to 25 m and 120 m.

The model first simulates 200,000 dispersal events, as shown in figure *. Then these events are sorted into 50x50 m bins around the source, and the proportion of seed or pollen dispersing to each of these bins is calculated


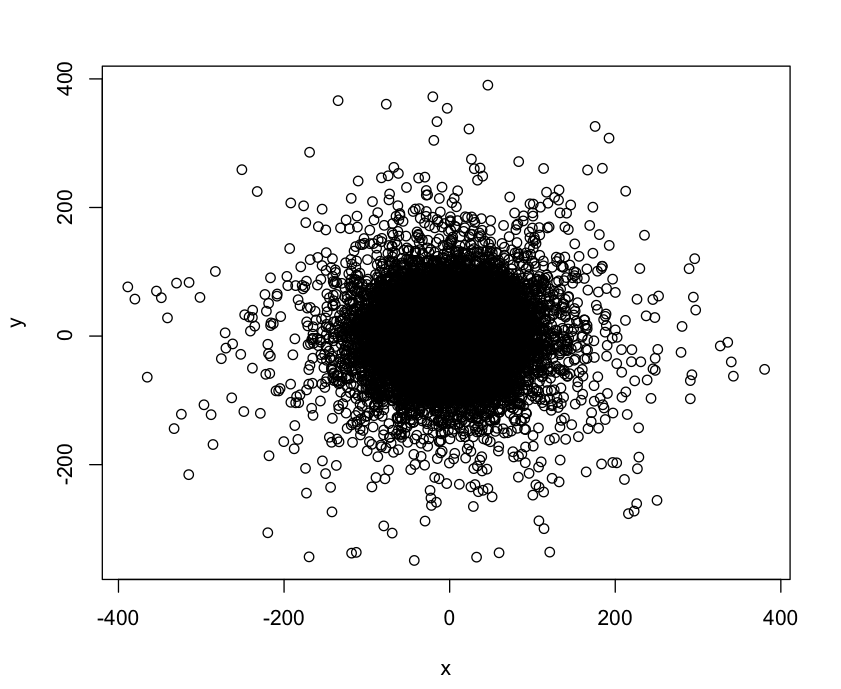


**Disturbance:**

|  | Seedling survival | Sapling survival | Small tree survival | Med. tree survival | Large tree survival |
| --- | --- | --- | --- | --- | --- |
| Simple (SD) | 0.3 | 0.3 | 0.3 | 0.3 | 0.3 |
| Canopy (CD) | 0.3 | 0.15 | 0.5 | 0.1 | 0.2 |
| Understory (UD) | 0.1 | 0.15 | 0.3 | 0.7 | 0.9 |

In the simple disturbance scenario, all life stages within the affected patch have the same probability of survival. The canopy disturbance scenario mimics canopy fires in which moderate sized individuals are more likely to die than seedlings or large adults. The understory disturbance scenario mimics ground fires in which juvenile mortality is especially high. In ground fires there can be 29-98% mortality of Ponderosa pine seedlings and 50-70% mortality of saplings, depending on heterogeneity in fire intensity (Bailey and Covington 2002).

Disturbances had either a 15 year average return interval (probability in single year = 0.06667) or a 25 year average return interval (probability in single year = 0.04).

1. Kroiss SJ, H. R. Lambers J. Recruitment limitation of long-lived conifers: implications for climate change responses. 2015;

2. Krannitz PG, Duralia TE. Cone and seed production in Pinus ponderosa: a review. West North Am Nat. 2004;64: 208–218.

3. Burns RM, Honkala BH. Silvics of North America. Washington DC: USDA Forest Service; 1990.

4. McDonald PM. Estimating seed crops of conifer and hardwood species. Can J For Res. 1992;22: 832–838.

5. Latta RG, Linhart YB, Fleck D, Elliot M. Direct and Indirect Estimates of Seed Versus Pollen Movement Within a Population of Ponderosa Pine. Evolution. 1998;52: 61. doi:10.2307/2410920

6. Lesser MR, Jackson ST. Contributions of long-distance dispersal to population growth in colonising *Pinus ponderosa* populations. Nathan R, editor. Ecol Lett. 2013;16: 380–389. doi:10.1111/ele.12053

7. Robledo-Arnuncio JJ, Gil L. Patterns of pollen dispersal in a small population of Pinus sylvestris L. revealed by total-exclusion paternity analysis. Heredity. 2005;94: 13–22. doi:10.1038/sj.hdy.6800542
