# Supplementary material for: Simulating the Interacting Effects of Intraspecific Variation, Disturbance, and Competition on Climate-Driven Range Shifts in Trees
Source: PLoS One. 2015 Nov 11;10(11):e0142369. doi: 10.1371/journal.pone.0142369 (PMC4641630; doi:10.1371/journal.pone.0142369)
Supplement: S3 File — (DOCX) [file pone.0142369.s003.docx]

**S3: Stable size-class distribution for baseline model**

Size distribution at 150, 250, and 350 years after initial conditions are established, with no climate change. The mean numbers in each class are stable after 150 years.

| Size class | Time step (years) | | | | | |
| --- | --- | --- | --- | --- | --- | --- |
|  | 150 | | 250 | | 350 | |
|  | mean | range | mean | range | Mean | range |
| Seedlings | 276.72 | 94 - 474 | 276.73 | 98 - 474 | 276.62 | 73 - 469 |
| Saplings | 49.94 | 8 - 116 | 49.89 | 7 - 115 | 49.96 | 9 - 119 |
| Small trees | 20.33 | 2 - 46 | 20.32 | 1 - 48 | 20.33 | 3 - 49 |
| Medium trees | 10.8 | 0 - 31 | 10.78 | 0 - 31 | 10.78 | 0 - 28 |
| Large trees | 10.1 | 0 - 21 | 10.12 | 2 - 21 | 10.11 | 1 - 20 |
