# Supplementary material for: Simulating the Interacting Effects of Intraspecific Variation, Disturbance, and Competition on Climate-Driven Range Shifts in Trees
Source: PLoS One. 2015 Nov 11;10(11):e0142369. doi: 10.1371/journal.pone.0142369 (PMC4641630; doi:10.1371/journal.pone.0142369)
Supplement: S4 File — (DOCX) [file pone.0142369.s004.docx]

**S4: Occupation of climate 3 by LE and climate 4 by HE prior to climate change for all model variants.**

Table showing the number of adult LE individuals occupying climate 3 and HE individuals occupying climate 4 prior to climate change for each model variant. The numbers vary depending on colonization (affected by dispersal and fecundity) and recruitment opportunities (affected by disturbance and relative fitness).

|  | Climate 4 LE | | | Climate 3 HE | | |
| --- | --- | --- | --- | --- | --- | --- |
| Model | Mean | Min | Max | Mean | Min | Max |
| Base | 4.6 | 3 | 8 | 8.8 | 5 | 12 |
| SD | 27.6 | 19 | 37 | 23.5 | 12 | 33 |
| NH | 2546.9 | 2331 | 2723 | n/a | n/a | n/a |
| HNW | 72.7 | 52 | 93 | 2.1 | 0 | 5 |
| CD | 24.3 | 18 | 34 | 27.5 | 12 | 45 |
| UD | 9.5 | 5 | 11 | 10.9 | 5 | 19 |
| LNW | 0 | 0 | 0 | 72.7 | 45 | 105 |
| LTS | 4.2 | 2 | 7 | 4.7 | 3 | 10 |
| L | 13.1 | 7 | 17 | 14.1 | 8 | 21 |
| S | 1.2 | 0 | 4 | 1.5 | 0 | 3 |
| SD_25 | 24.6 | 13 | 38 | 20.3 | 10 | 41 |
| SD_HF | 30.9 | 19 | 49 | 35.7 | 27 | 47 |
| SD_HF_S | 10.5 | 5 | 18 | 11.8 | 7 | 18 |
| SD_L | 37.1 | 24 | 49 | 42 | 28 | 72 |
| SD_S | 8.3 | 4 | 20 | 7.9 | 5 | 17 |
| NH_L | 3836.4 | 3470 | 4093 | 0 | 0 | 0 |
| HNW_SD | 106 | 84 | 128 | 3.8 | 0 | 6 |
| HNW_L | 117.9 | 99 | 140 | 4.2 | 1 | 9 |
| HNN2 | 403.4 | 322 | 544 | 0 | 0 | 0 |
| HNN3 | 6.3 | 2 | 10 | 6.1 | 4 | 9 |
| CD_25 | 27.4 | 13 | 42 | 25.9 | 10 | 45 |
| CD_L | 49.4 | 36 | 69 | 38.6 | 25 | 48 |
| CD_S | 8.5 | 3 | 18 | 9 | 4 | 17 |
| UD_25 | 7.9 | 5 | 13 | 9.3 | 5 | 19 |
| UD_L | 18 | 12 | 24 | 21.7 | 15 | 30 |
| UD_S | 3.2 | 0 | 7 | 2 | 0 | 4 |
