# Supplementary material for: Simulating the Interacting Effects of Intraspecific Variation, Disturbance, and Competition on Climate-Driven Range Shifts in Trees
Source: PLoS One. 2015 Nov 11;10(11):e0142369. doi: 10.1371/journal.pone.0142369 (PMC4641630; doi:10.1371/journal.pone.0142369)

**S5: Approach to stable equilibrium post climate change for baseline model, SD, NH, and HNW.**

Figure A: Time to equilibrium, baseline model. Changes in W allele frequency and extinction/colonization lag for the two species over a 3000 years at 500 year intervals, starting at the end of the climate change period. First column EQ: equilibrium expected to be reached by the baseline model.


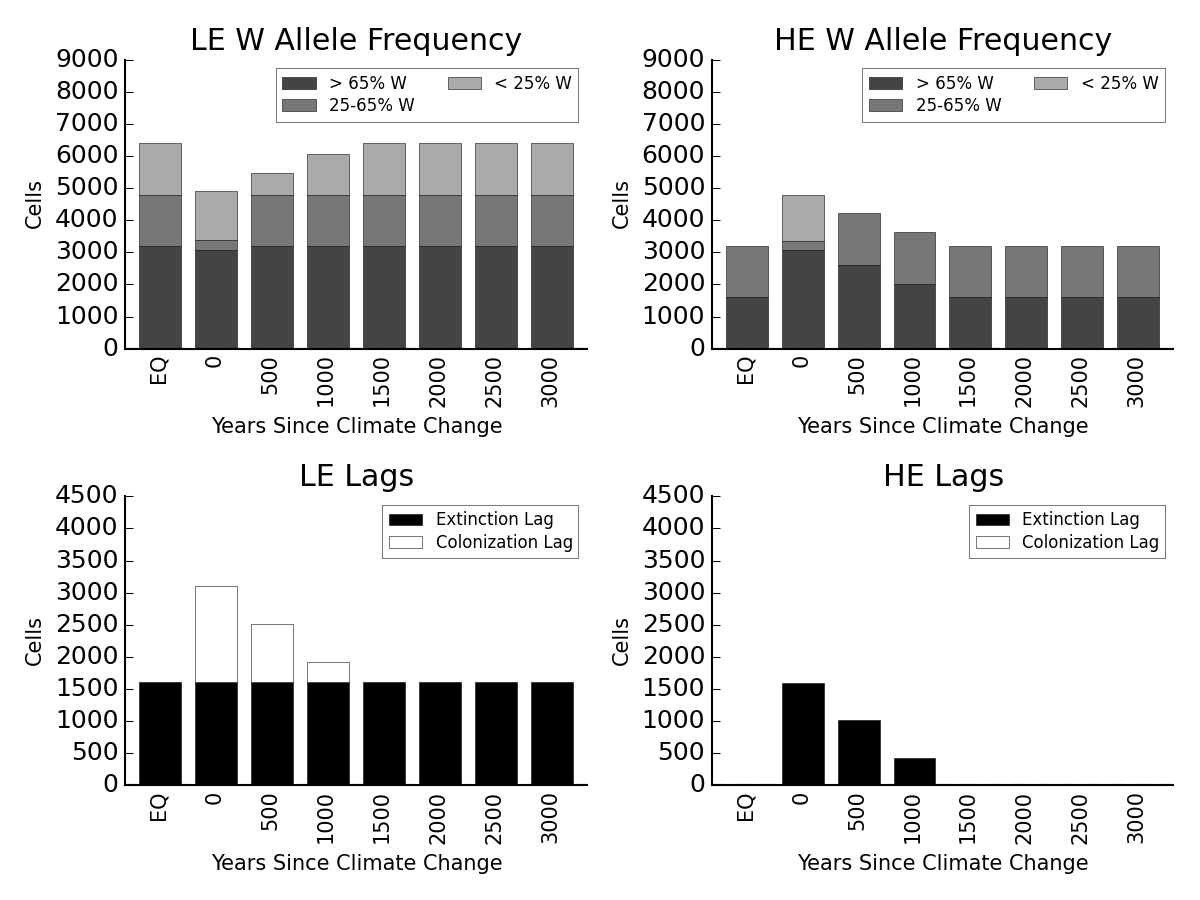


Figure B:Time to equilibrium, simple disturbance model. Changes in W allele frequency and extinction/colonization lag for the two species over a 3000 years at 500 year intervals, starting at the end of the climate change period. Notice that actual LE patches >65% W are less common at equilibrium than expected in baseline model (EQ); recolonization of disturbed patches is slow in sub-optimal climate 7 area. Notice also that equilibrium is reached more quickly.


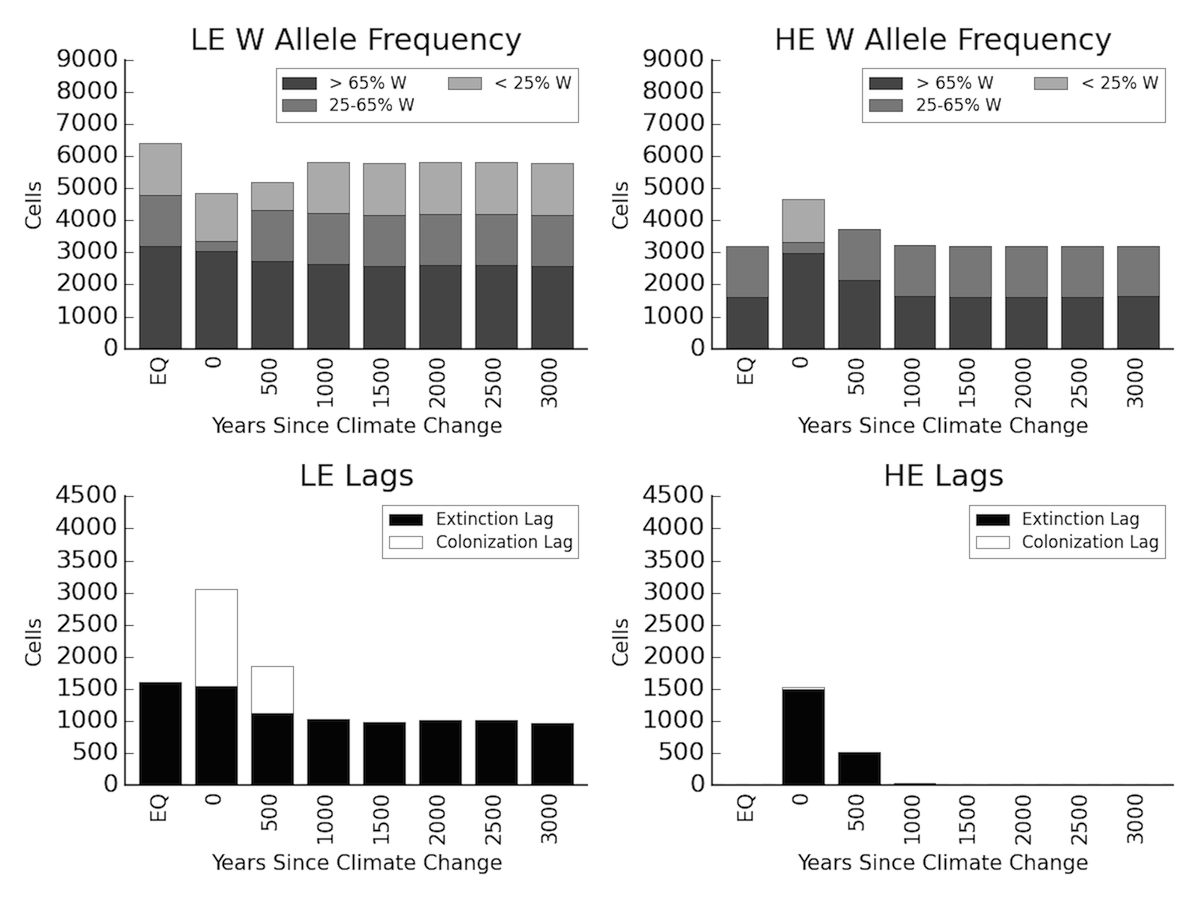


Figure C:Time to equilibrium for model with an HE species that has no genetic variation and broad environmental tolerances. Changes in W allele frequency and extinction/colonization lag for the two species over a 3000 years at 500 year intervals, starting at the end of the climate change period. Notice that equilibrium is reached more quickly than for baseline model. EQ: equilibrium expected for baseline model.


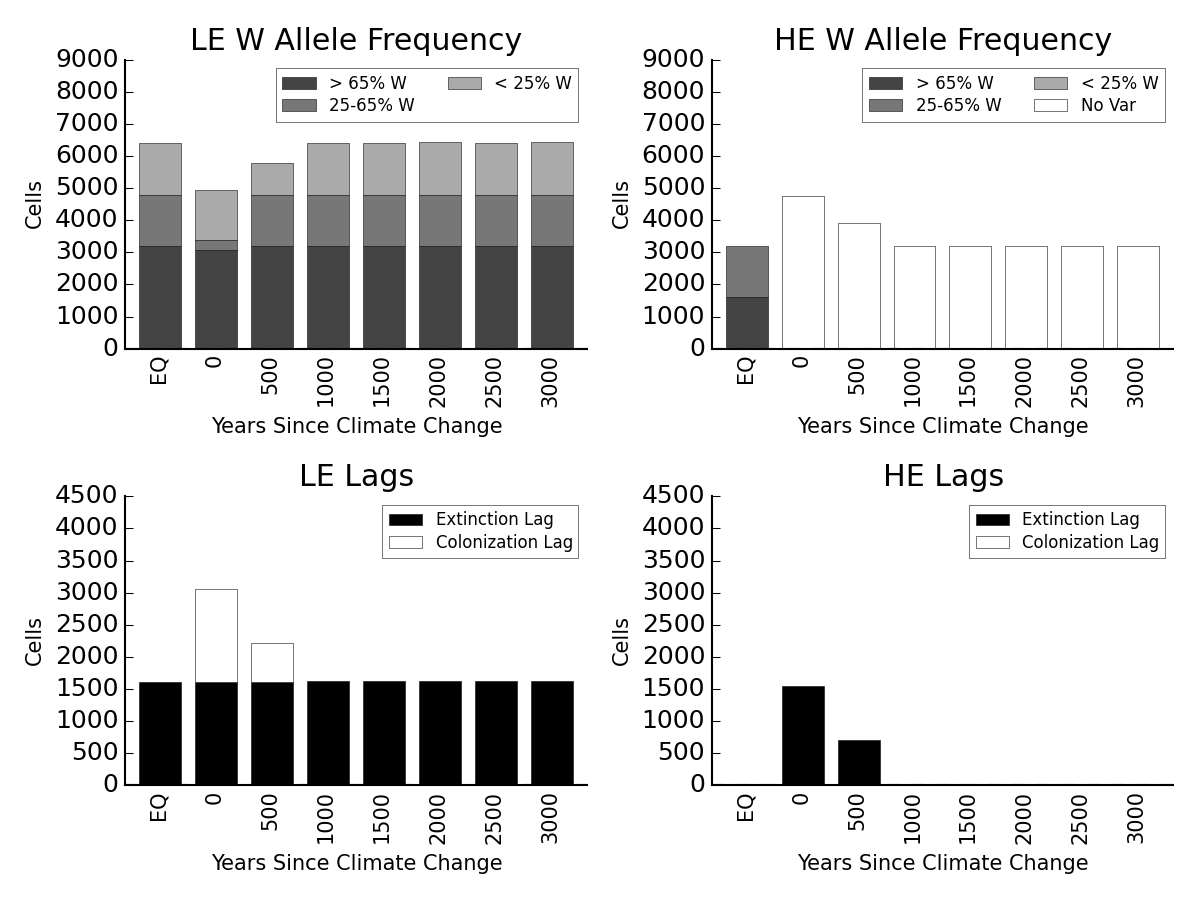


Figure D: Time to equilibrium for LE with no competing HE species. Changes in W allele frequency and extinction/colonization lag for the two species over a 4000 years at 500 year intervals starting at the end of the climate change period. Notice that it takes longer to reach full equilibrium, and that the area colonized by populations with <25% W alleles is greater than in the baseline equilibrium (EQ), because without an HE competitor LE can survive in the sub-optimal climate 2, but it takes time to reach this more distant climate band.
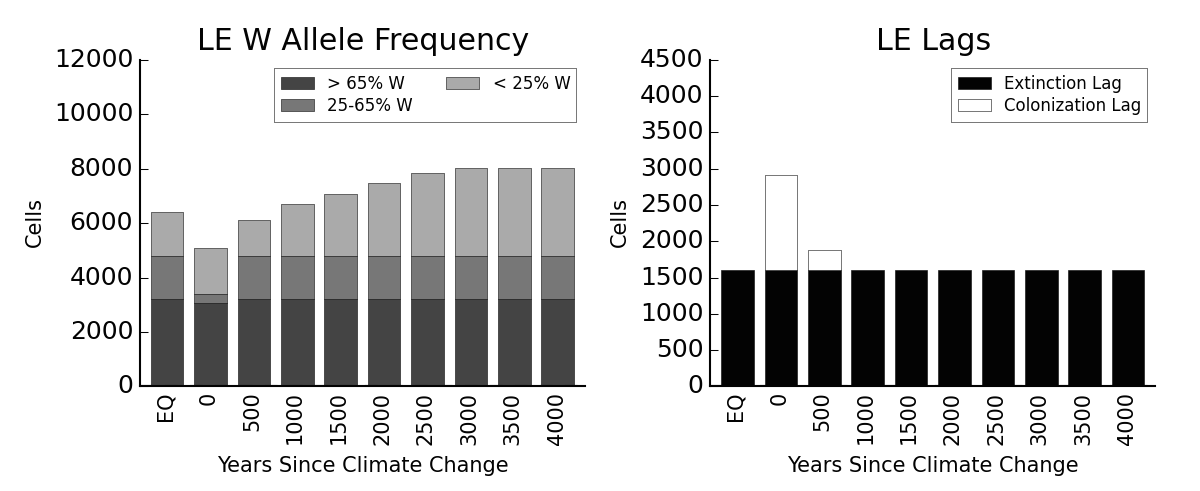

Supplement: S5 File — (DOCX) [file pone.0142369.s005.docx]
