# Supplementary material for: Simulating the Interacting Effects of Intraspecific Variation, Disturbance, and Competition on Climate-Driven Range Shifts in Trees
Source: PLoS One. 2015 Nov 11;10(11):e0142369. doi: 10.1371/journal.pone.0142369 (PMC4641630; doi:10.1371/journal.pone.0142369)

**S6:** **Figures showing results of additional model variants, including interactions between dispersal and fecundity or disturbance type, and the effects of genetic variation.**

Figure A: Effects of changing fecundity vs. dispersal distance. EQ: equilibrium reached by the baseline model; Base: baseline model; SD: simple disturbance; SD_S: SD, short dispersal; SD_L: SD, long dispersal; SD_HF: SD, high fecundity (4x more seed produced); SD_HF_S: SD, high fecundity, short dispersal.


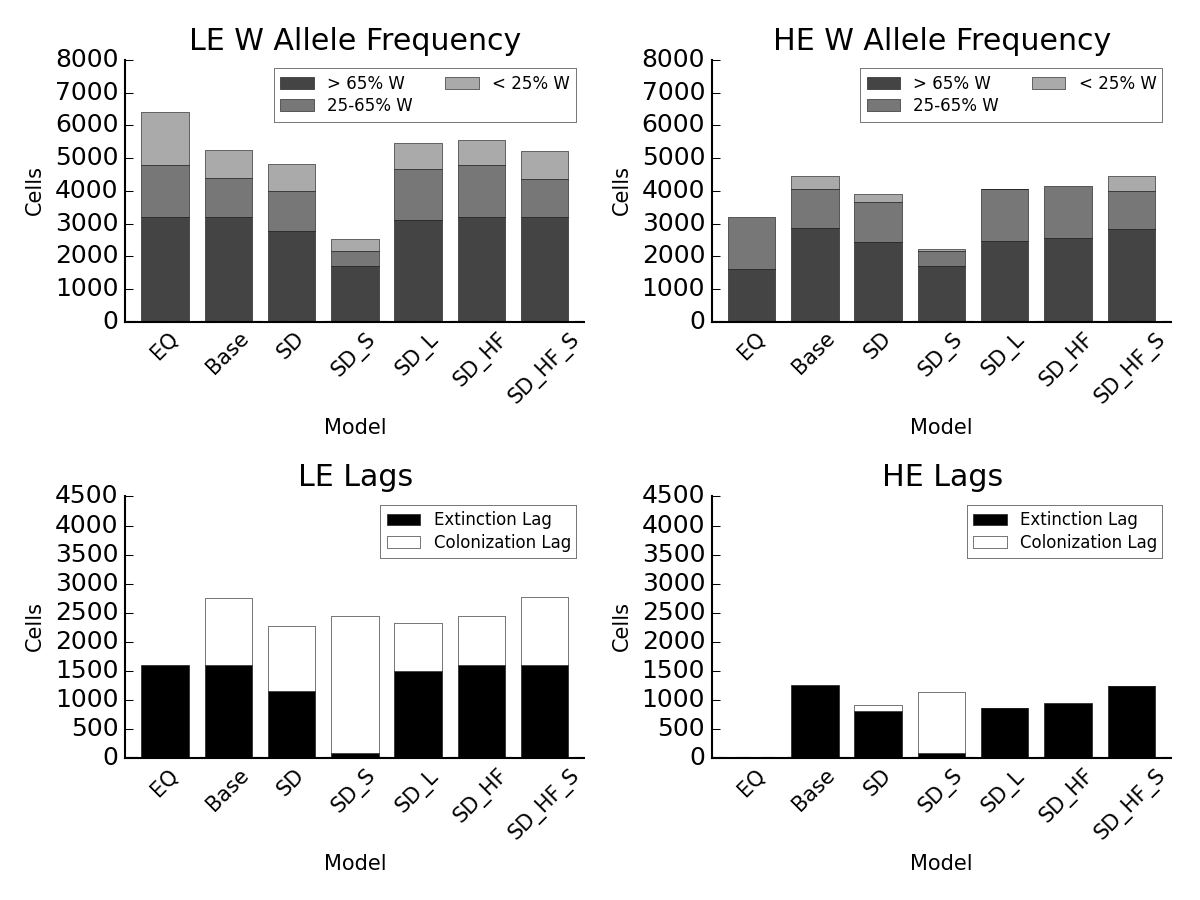


Figure B: Interaction between dispersal distance and simple vs. canopy disturbance. EQ: equilibrium reached by the baseline model; Base: baseline model; SD: simple disturbance; CD: canopy disturbance; SD_S: SD, short dispersal; CD_S: CD, short dispersal; SD_L: SD, long dispersal; CD_L: CD, long dispersal.


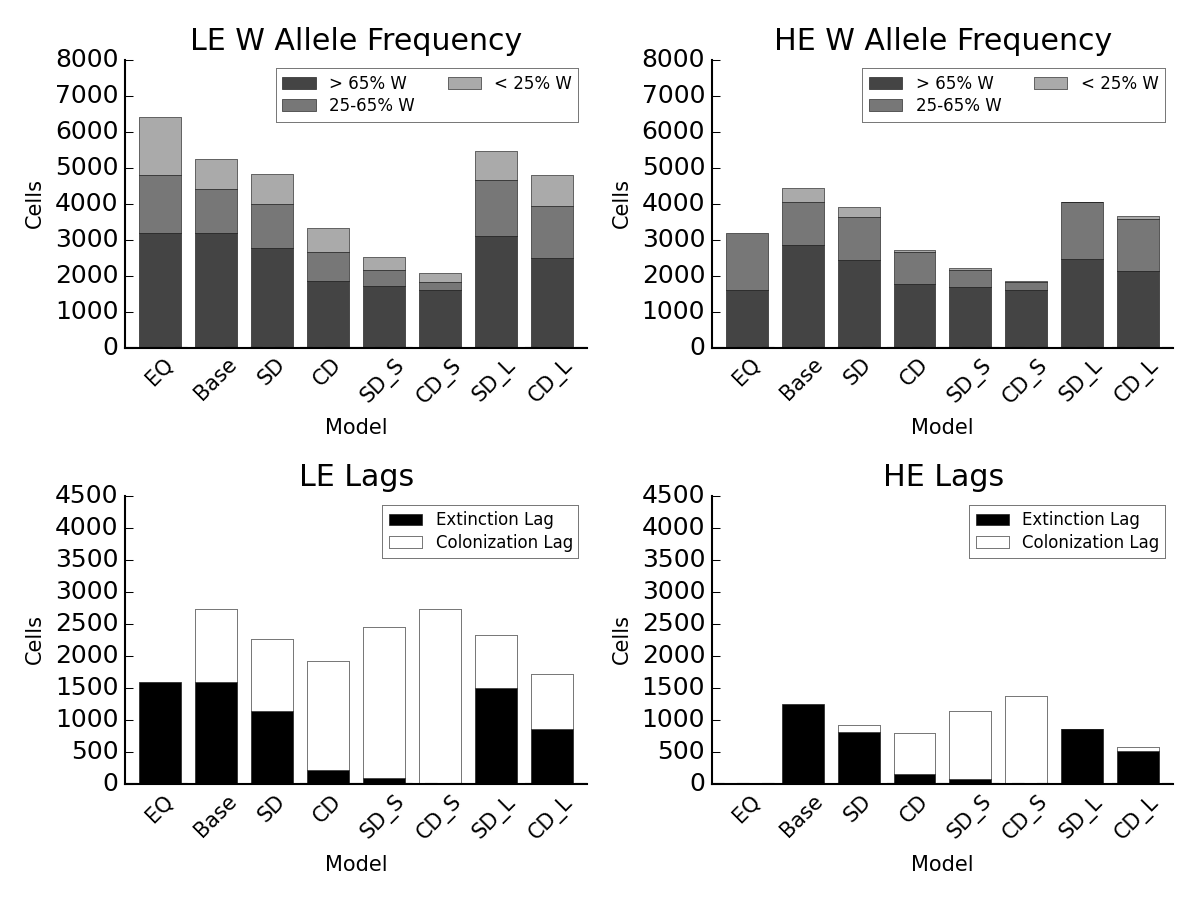


Figure C: Interaction between dispersal distance and simple vs. understory disturbance. EQ: equilibrium reached by the baseline model; Base: baseline model; SD: simple disturbance; UD: understory disturbance; SD_S: SD, short dispersal; UD_S: UD, short dispersal; SD_L: SD, long dispersal; UD_L: UD, long dispersal.


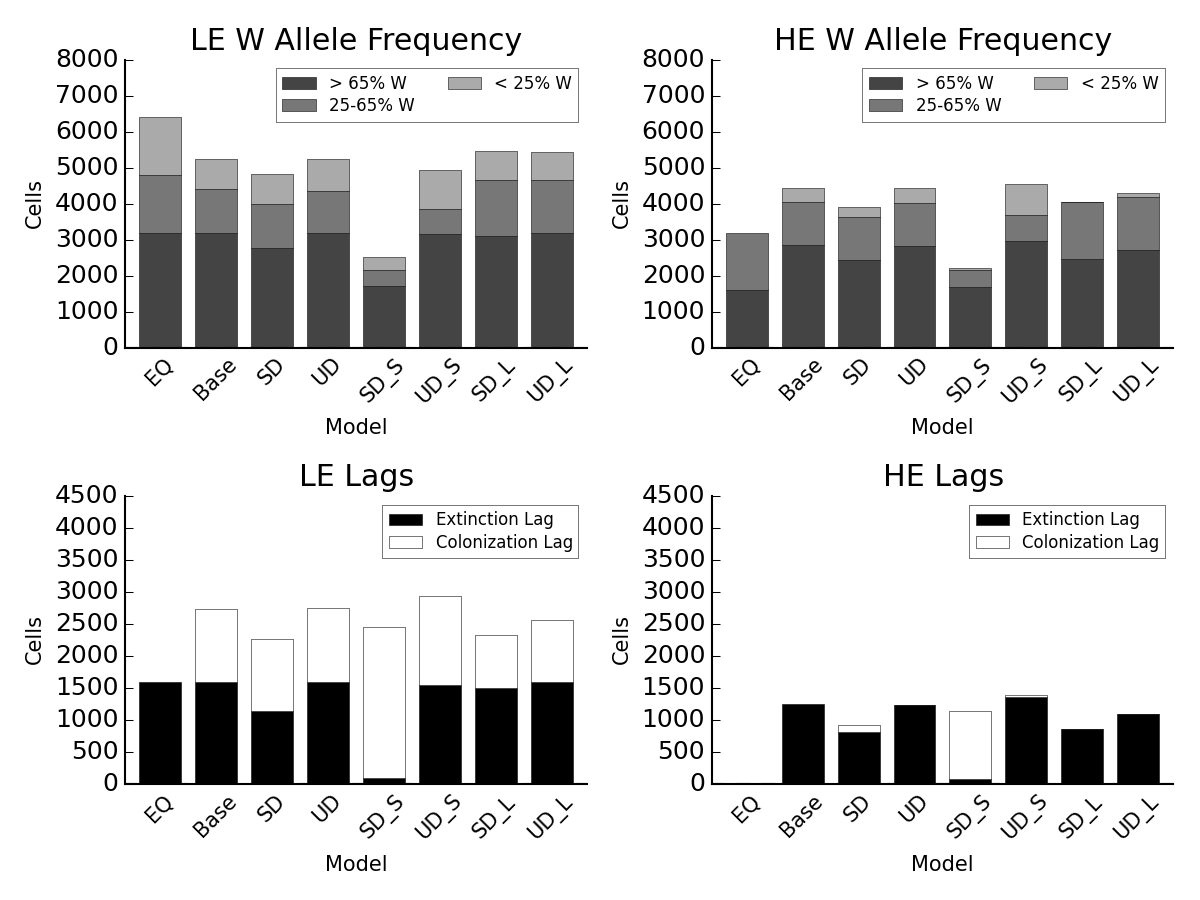


Figure D: Genetic variation’s effects on low-elevation species spread. EQ: equilibrium reached by the baseline model; Base: baseline model; LNW: LE species single genotype with wide tolerance (optimum at climate 5); HNW: HE species single genotype with wide tolerance (optimum at climate 2); HNW_SD: HNW, simple disturbance.


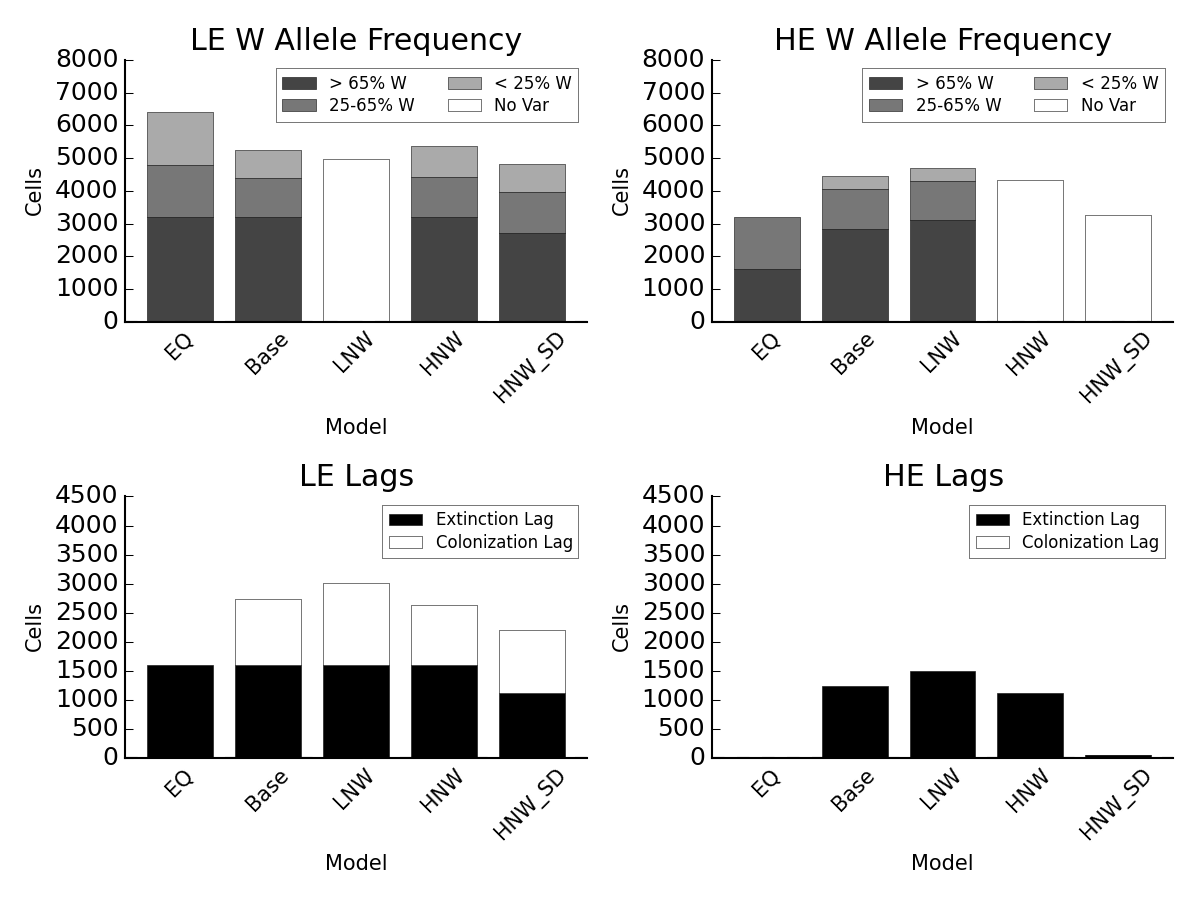

Supplement: S6 File — (DOCX) [file pone.0142369.s006.docx]
